# Pathogenic tau recruits wild-type tau into brain inclusions and induces gut degeneration in transgenic SPAM mice

**DOI:** 10.1038/s42003-022-03373-1

**Published:** 2022-05-12

**Authors:** Yuxing Xia, Stefan Prokop, Brach M. Bell, Kimberly-Marie M. Gorion, Cara L. Croft, Lith Nasif, Guilian Xu, Cara J. Riffe, Alyssa N. Manaois, Kevin H. Strang, Stephan S. Quintin, Giavanna Paterno, Malú Gámez Tansey, David R. Borchelt, Todd E. Golde, Benoit I. Giasson

**Affiliations:** 1 Department of Neuroscience, College of Medicine, University of Florida, Gainesville, FL 32610 USA; 2 Center for Translational Research in Neurodegenerative Disease, College of Medicine, University of Florida, Gainesville, FL 32610 USA; 3 McKnight Brain Institute, College of Medicine, University of Florida, Gainesville, FL 32610 USA; 4 Department of Pathology, College of Medicine, University of Florida, Gainesville, FL 32610 USA

**Keywords:** Alzheimer's disease, Neurodegeneration

## Abstract

Pathological tau inclusions are neuropathologic hallmarks of many neurodegenerative diseases. We generated and characterized a transgenic mouse model expressing pathogenic human tau with S320F and P301S aggregating mutations (SPAM) at transgene levels below endogenous mouse tau protein levels. This mouse model develops a predictable temporal progression of tau pathology in the brain with biochemical and ultrastructural properties akin to authentic tau inclusions. Surprisingly, pathogenic human tau extensively recruited endogenous mouse tau into insoluble aggregates. Despite the early onset and rapid progressive nature of tau pathology, major neuroinflammatory and transcriptional changes were only detectable at later time points. Moreover, tau SPAM mice are the first model to develop loss of enteric neurons due to tau accumulation resulting in a lethal phenotype. With moderate transgene expression, rapidly progressing tau pathology, and a highly predictable lethal phenotype, the tau SPAM model reveals new associations of tau neurotoxicity in the brain and intestinal tract.

## Abstract (web summary)

A mouse model with a predictable pathogenic tau protein progression is presented, enabling further investigation into tau pathology in neurodegenerative diseases.

## Introduction

Alzheimer’s disease (AD) is the most common neurodegenerative disorder and currently affects more than 25 million people worldwide^1^. The primary clinical symptoms of AD are progressive memory loss and cognitive decline. Neuropathologically, AD is defined by two major hallmarks: Aβ peptides in senile plaques and hyperphosphorylated tau in neurofibrillary tangles (NFT)^2^. Aside from AD, tau is associated with a heterogeneous group of neurodegenerative disorders, including frontotemporal dementia (FTD) and chronic traumatic encephalopathy^3,4^ and mutations in the microtubule-associated protein Tau (*MAPT*) gene cause familial FTD^3,5^. In tauopathies like AD and FTD, the “tau hypothesis” proposes that tau protein is a major toxic factor driving disease progression and neurodegeneration^6^.

Transgenic murine models that express tau mutations such as P301L and P301S have been used to model tau pathology that is found in AD, FTD, and other tauopathies^7^. However, many of these models are less than ideal due to high levels of transgene overexpression and inconsistent pathology or major sex differences^8,9^. To create a robust and reproducible mouse model of tauopathy, we expressed pathogenic tau with S320F and P301S-aggregating mutations (SPAM) under the mouse prion promoter^10^ and identified a line of tau SPAM mice that expresses pathogenic human tau below endogenous mouse tau levels. During our screening of tau missense mutations, we confirmed that pathogenic tau SPAM uniquely promotes aggregation to form hyperphosphorylated tau inclusions in vivo^11–14^. The tau mutations P301S and S320F have been reported to cause frontotemporal dementia (FTD) with Pick bodies^15–17^ in affected individuals. This approach of combining multiple disease- associated mutations in transgenic models has been used to model tau pathology^18–20^. Additionally, commonly used 3xTg-AD and 5xfAD models contain three and five different AD familial mutations, respectively^21,22^. The newly generated SPAM model of tauopathy exhibits accelerated tau pathology. Robust hyperphosphorylated tau inclusions appear in the cortex and hippocampus at 2 months and rapidly progress throughout the brain at 4–6 months.

Intestinal symptoms such as constipation^23,24^ are a common feature of chronic neurological diseases like AD and enteric dysfunction associated with neuronal loss is linked to aging and dementia^25–27^. Some early evidence suggests that tau may potentially be involved in enteric dysfunction: tau protein is expressed in human enteric neurons^28^ and can be found in sigmoid colons of AD and FTD patients^29^. Interestingly, we found that the tau SPAM mice express mutant tau in neurons of the myenteric plexus, resulting in increased tau phosphorylation at multiple sites, including pThr205, pThr231, pSer396, and pSer404. This accumulation of phosphorylated tau in enteric neurons of SPAM mice leads to significant neuronal loss and a lethal intestinal phenotype at 6 months. Overall, the SPAM mouse model robustly displays neuronal tau pathology found in AD and FTD, and serves as a platform to study tau-associated neurotoxicity and degeneration in both brain and gut, and additionally can be used for rapid therapeutic screening.

## Results

### The tau SPAM model develops robust NFT-like inclusions similar to late AD Braak stages

**Fig. 1:**
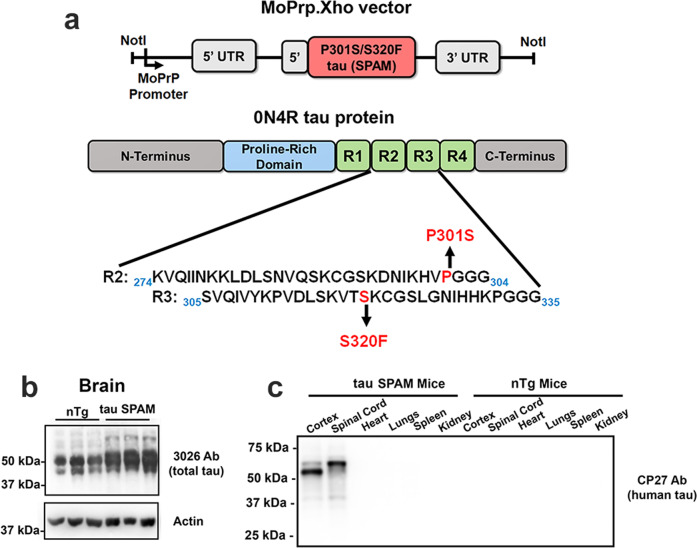
Generation of transgenic mice that express pathogenic tau with the S320F and P301S-aggregating mutations (SPAM). **a** Pathogenic tau with the S320F and P301S-aggregating mutations (SPAM) in the 0N4R human tau isoform is expressed under the mouse prion promoter was cloned in the MoPrP.Xho vector as shown in the diagram. **b** Western blot analysis with total tau antibody 3026 of total brain lysates (*N* = 3) from 2-month-old nTg and tau SPAM mice. 10 μg of protein was loaded per lane. Actin was used as a loading control. Tau SPAM mice express total tau at 1.7X-fold compared with endogenous mouse tau levels in nTg mice. The relative molecular weight markers are shown on the left. **c** Western blot analysis with antibody CP27 specific for human tau showing relative tau expression in major tissue (cortex, spinal cord, heart, lungs, spleen, and kidney) from 6-month-old tau SPAM and nTg mice. 10 μg of protein was loaded per lane. Human tau expression is primarily found in the brain and spinal cord. The shift in tau with reduced mobility is due to hyperphosphorylation associated with aggregation. The relative molecular mass markers are shown on the left.

To create a robust and reproducible mouse model of tau pathology, we generated new transgenic mice that express the human tau S320F and P301S-aggregating mutations under the pan-neuronal mouse prion promoter, MoPrP^10^ (Fig. 1a). Five tau transgenic founders were obtained, and four transgenic lines were confirmed to have germline transmission and expression. We focused on the By line of tau SPAM mice (subsequently referred to as tau SPAM mice) due to its consistent and robust tau pathology and the development of unique phenotypes. The integration site was mapped to a region in chromosome 7 and the genes altered do not significantly affect CNS or intestinal phenotypes (see “Materials and methods” for more details), but we cannot completely exclude indirect effects or interactions.

Both human and mouse tau RNA levels were comparable between tau SPAM mice at ages of 2, 4, and 6 months for both sexes (Supplemental Fig. 1). Using a total tau antibody 3026, we found that tau SPAM mice express low levels of pathogenic tau protein (1.7X overall tau levels compared with nTg mice or 0.7X human SPAM tau relative to endogenous mouse tau) as measured by western blotting (Fig. 1b; Supplemental Fig. 1c). Pathogenic tau is primarily expressed in the brain and spinal cord, with little to no expression in peripheral tissues such as heart, lungs, spleen, and kidney on immunoblotting with CP27 antibody specific for human tau (Fig. 1c). However, very low tau expression was also found in intestinal tissues (see Fig. 10a).

**Fig. 2:**
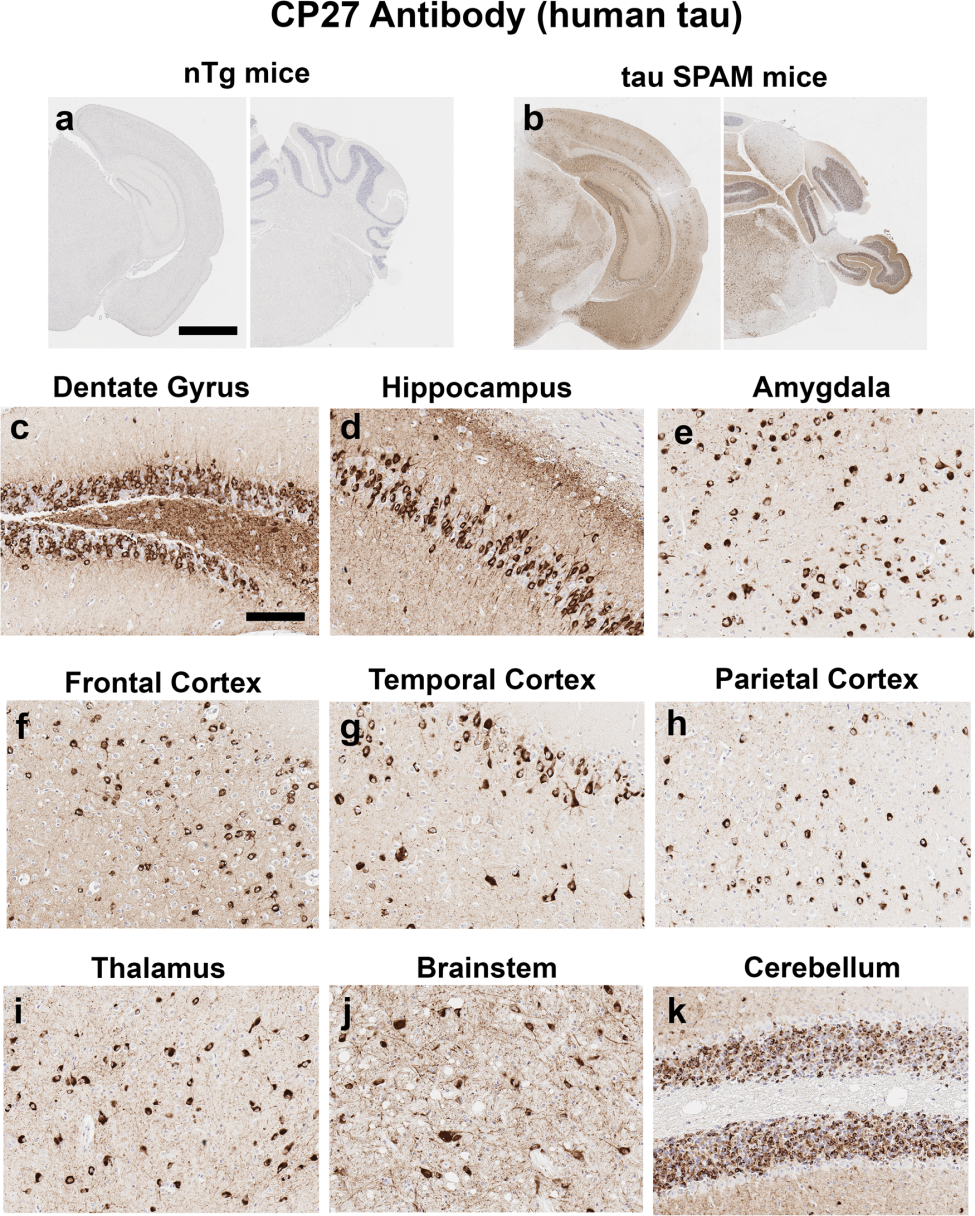
Tau SPAM mice develop robust NFT-like tau inclusions throughout most of the brain neuroaxis, particularly in the hippocampus and cortex. **a**, **b** IHC with antibody CP27 specific for human tau does not stain brain tissue from nTg mice, but reveals a high density of tau inclusions in 6-month-old tau SPAM mice. Scale bar = 1.333 mm. Higher-magnification pictures show abundant perikaryal NFT-like tau inclusions in all major areas of the brain, including (**c**) dentate gyrus, (**d**) hippocampus, (**e**) amygdala, (**f**) frontal cortex, (**g**) temporal cortex, (**h**) parietal cortex, (**i**), thalamus, (**j**) brainstem, and (**k**) granular layer of the cerebellum. Tissue sections were counterstained with hematoxylin. Scale bar = 100 μm.

At 6 months of age, tau SPAM mice present with widespread NFT-like tau inclusions detected immunohistochemically with the CP27 antibody (Fig. 2) throughout the neuroaxis, including the hippocampus, entorhinal areas, visual, parietal, and temporal brain cortices, amygdala, brainstem, dentate gyrus, thalamus, and granular layer of cerebellum. Morphologically, these inclusions resemble perikaryal NFT, but neuropil threads can also be found in different brain regions.

### Tau SPAM mice accumulate Sarkosyl-insoluble tau aggregates hyperphosphorylated at multiple residues

**Fig. 3:**
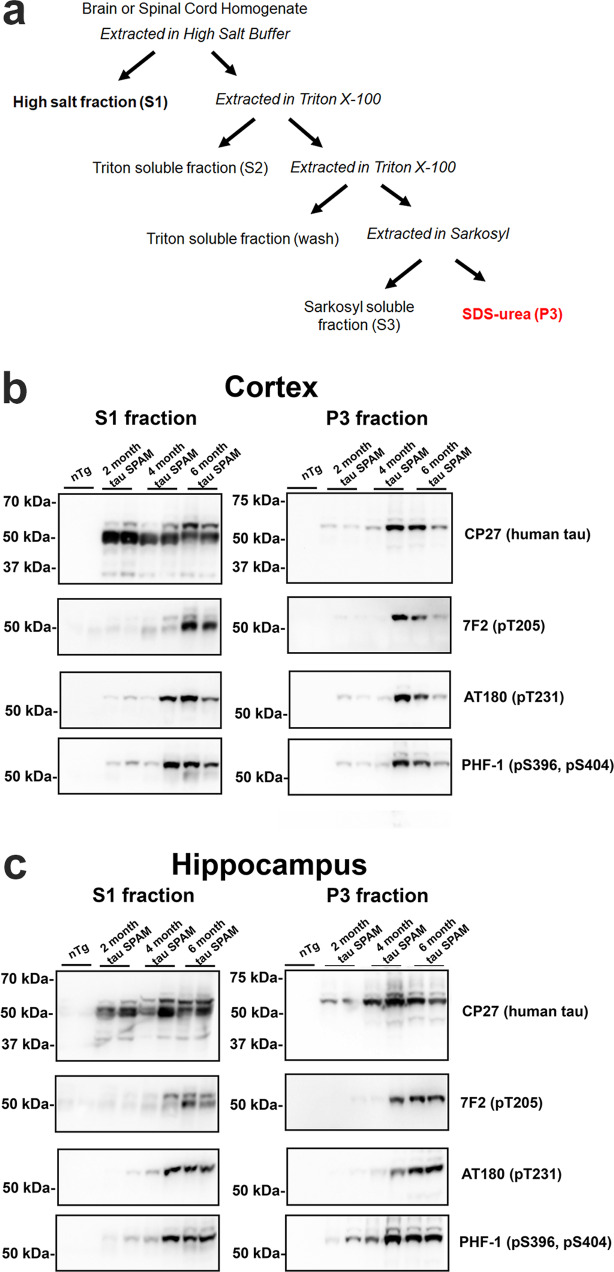
Temporal accumulation of aggregated, hyperphosphorylated tau in tau SPAM mice. **a** Summary diagram of biochemical, sequential extraction used. The S1 fraction contains primarily soluble tau and the P3 fraction contains Triton X-100 and Sarkosyl-insoluble tau. **b** Brain cortex and (**c**) hippocampus from tau SPAM mice at 2, 4, and 6 months of age were sequentially extracted. The soluble fraction (S1) and pellet fraction (P3) were resolved by immunoblotting with CP27 antibody specific for human tau and tau phospho-specific antibody 7F2, (pThr205), AT180 (pThr231), or PHF1 (pSer396/pSer404). The relative molecular mass markers are shown on the left.

By performing sequential detergent extraction, Triton-insoluble and Sarkosyl-insoluble tau aggregates were biochemically isolated from brain lysates of SPAM mice at 2, 4, and 6 months of age (Fig. 3a). This procedure has been widely validated using human lysates from AD patients to isolate PHF-tau^30,31^. In the cortex, nearly all of the detergent-insoluble tau is hyperphosphorylated as reflected by the shift to apparent higher molecular mass from ~55 to 64 kDa on SDS-PAGE (Fig. 3b). In the hippocampus, there were also multiple higher molecular mass bands indicating higher levels of hyperphosphorylation at multiple sites (Fig. 3c), as this shift toward higher molecular weight is reversed by in vitro dephosphorylation (Supplemental Fig. 2).

The presence of higher molecular weight tau was confirmed using multiple phosphorylation-specific antibodies: 7F2 specific for tau phosphorylated at Thr205 (pThr205), AT180 specific for tau phosphorylated at Thr231, and PHF1 specific for tau phosphorylated at Ser396 and Ser404. All of these phosphorylation sites are classically hyperphosphorylated in late-stage AD^32–36^. Additionally, detergent-insoluble tau and phosphorylation levels in the P3 fraction rapidly increase with age from 2 to 4 to 6 months. Surprisingly, a significant amount of the total soluble tau even in the S1 fraction was phosphorylated and increased progressively with age from 2 to 6 months. By contrast, S2 and S3 fractions traditionally have lower levels of tau due to the extraction process and tau could be weakly detected in the S2 fractions (Supplemental Fig. 3), but was below detectable levels in the S3 fractions.

### Tau SPAM mice develop progressive, age-dependent hyperphosphorylated tau inclusions

In order to monitor the progression of tau pathology, brain sections from SPAM mice at 2, 4, and 6 months were stained with phosphorylation-specific antibodies. Antibodies 7F2, AT180, and PHF1 detect pThr205, pThr231, and pSer396/404 tau, respectively^33,34,37^. We quantified the density of tau inclusions immunohistologically in different brain areas, including hippocampus, cortex, amygdala, brainstem, and thalamus using Aperio algorithms (detailed in Materials and methods).

**Fig. 4:**
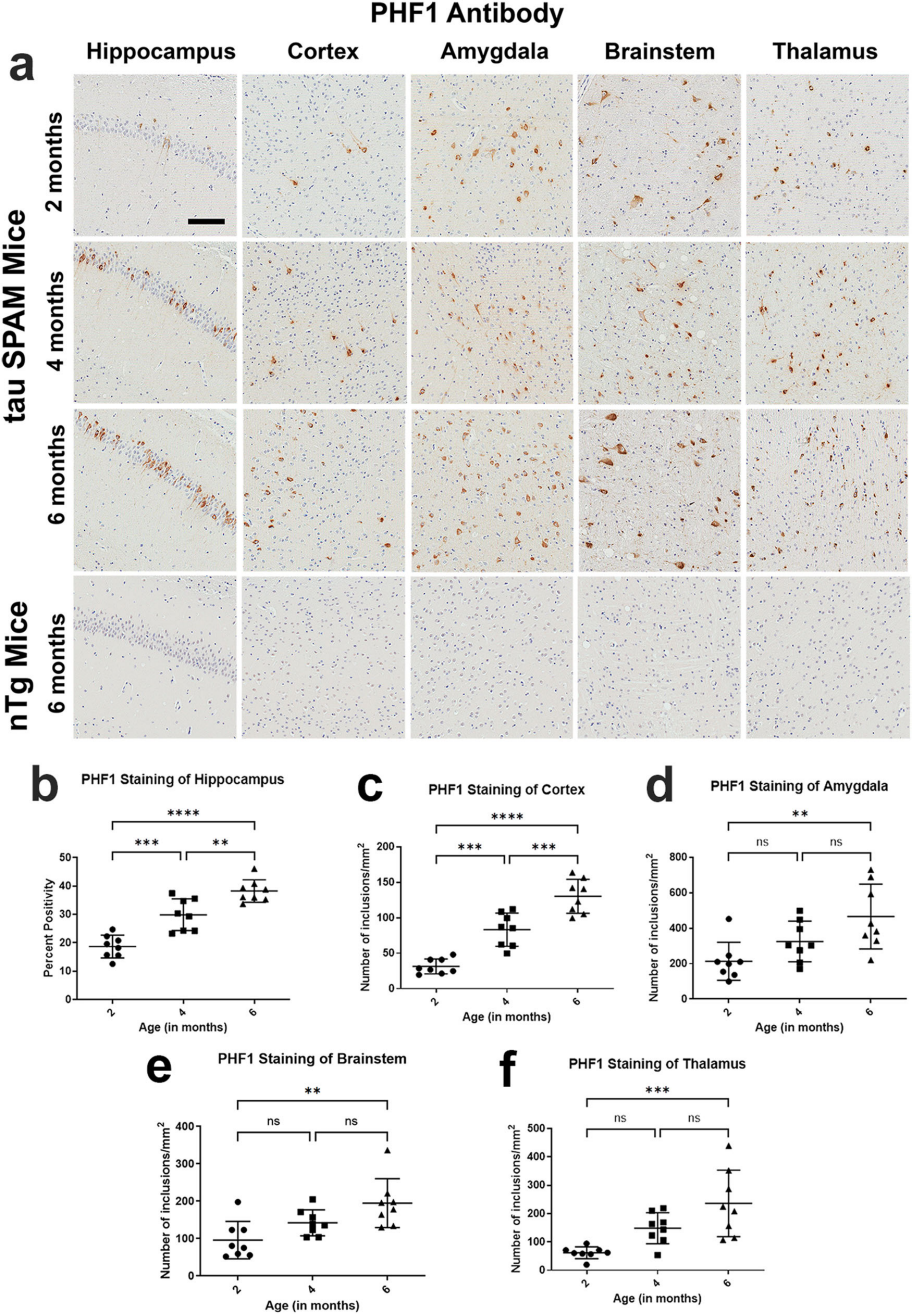
PHF1-positive phosphorylated tau inclusions progressively increase with age in tau SPAM mice. **a** Brain samples from tau SPAM mice at ages 2, 4, and 6 months were stained by IHC with PHF1 antibody specific for tau phosphorylated at S396 and S404. Tau inclusions were abundantly found in multiple brain areas as early as 2 months, but further increase with age. Scale bar = 100 μm. Tau inclusions progressively increased in the (**b**) hippocampus [one-way ANOVA, F (2, 21) = 36.75, *p* < 0.0001], (**c**) cortex [one-way ANOVA, F (2, 21) = 47.72, *p* < 0.0001], (**d**) amygdala [one-way ANOVA, F (2, 21) = 6.641, *p* = 0.0058], (**e**) brainstem [one-way ANOVA, F (2, 21) = 7.383, *p* = 0.0037], and (**f**) thalamus [one-way ANOVA, F (2, 21) = 10.60, *p* = 0.0007] of 2-, 4-, and 6-month-old mice (*N* = 8, 4 M, 4 F for each time point). Pathology was quantified with Aperio algorithms and compared using one-way ANOVA with Dunnett’s test. Error bars represent standard errors of the mean. ****p < 0.0001, ***p < 0.001, **p < 0.01, ns not statistically significant.

SPAM mice developed robust hyperphosphorylated tau inclusions within the hippocampus as early as 2 months (Fig. 4, and Supplemental Figs. 4, 5). NFT-like tau inclusions increased progressively by age from 2 to 6 months. At 6 months, phosphorylated tau inclusions were present in nearly all brain areas of cortex, hippocampus, amygdala, brainstem, and thalamus. In particular, the cortex showed significant age-dependent increase in tau-inclusion density and can be used to model widespread cortical pathology found in the endpoint of Braak stage V/VI. We did not observe major sex differences with respect to tau- inclusion density for all three phospho-antibodies in 6-month-old mice (Supplemental Fig. 6).

### Tau inclusions in tau SPAM mice exhibit hallmarks of human PHFs

**Fig. 5:**
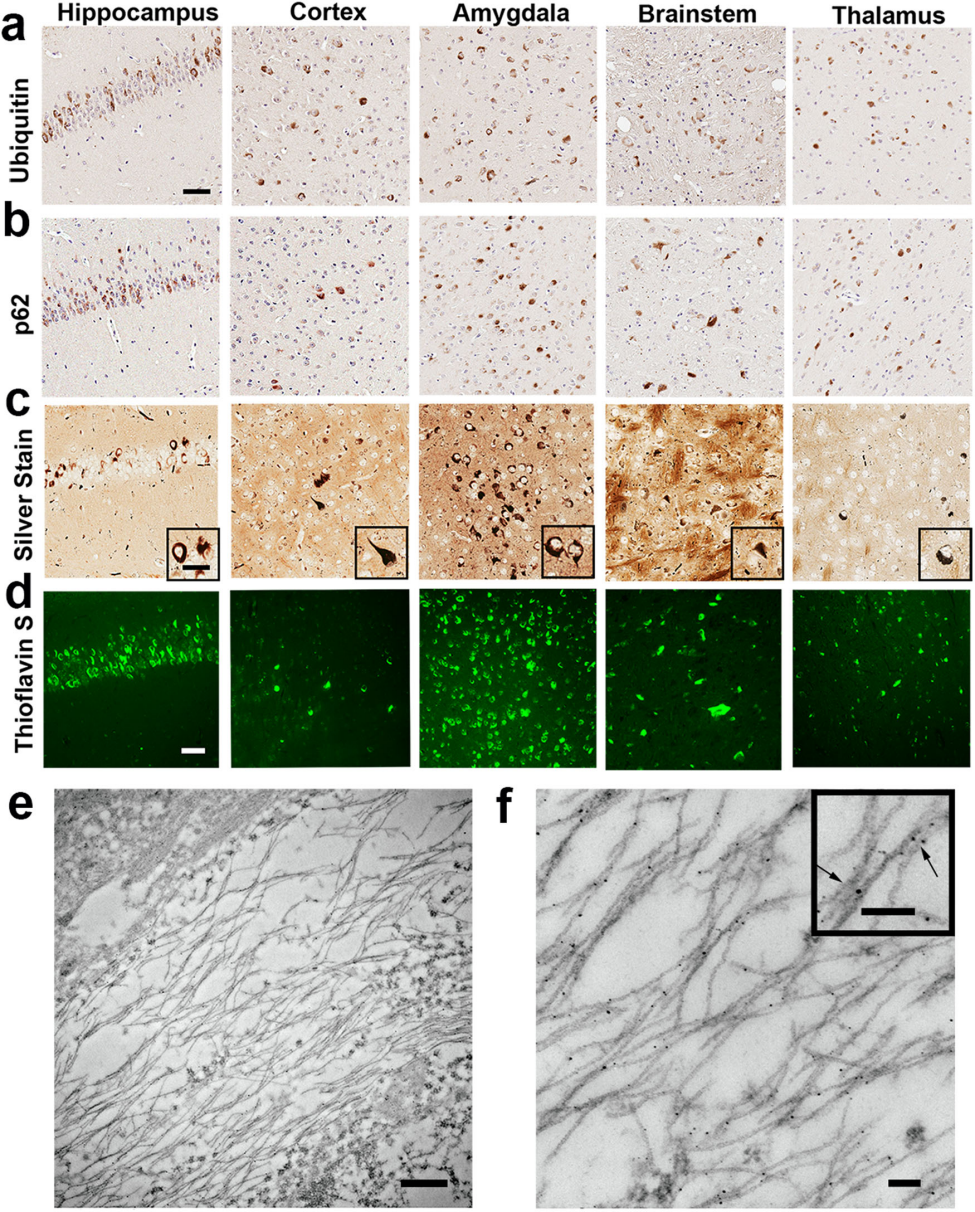
Histological and ultrastructural characterization of tau SPAM mice pathological tau inclusions. IHC staining of tau pathological inclusions with (**a**) anti-ubiquitin and (**b**) anti-p62/sequestosome-1. In 6-month-old tau SPAM mice, tau inclusions were also (**c**) argyophillic as stained with Campbell–Switzer silver stain and (**d**) readily labeled with Thioflavin S. Scale bars are 50 μm for main image and 25 μm for inset. **e** Immuno-EM analysis revealed that tau aggregates are composed of ultrastructural tau filaments as detected and labeled with 3026 antibody against total tau. Scale bar = 500 nm. **f** Magnified image shows pathogenic tau within straight filamentous structures. Scale bar = 100 nm for main image and inset.

To confirm the presence of true protein aggregates, brain areas with the most tau pathology were stained with antibodies against ubiquitin and p62 (Fig. 5a, b). Ubiquitin and p62 (sequestosome-1) are general markers for elevated protein degradation around tau aggregates^38–40^. Additionally, Thioflavin S or silver staining are gold standards in the field used to confirm protein aggregates with amyloid structure, which are key hallmarks of tau inclusions in tauopathies like AD^41–44^. In a 6-month-old tau SPAM mouse, Thioflavin-S-positive and argyrophilic inclusions were abundant in the hippocampus and amygdala (Fig. 5c, d). Other areas with significant tau aggregates included different cortical regions, thalamus, and brainstem. Despite the widespread presence of tau aggregates, neuron density within the cortex was not significantly different between tau SPAM mice and nTg mice at 6 months (Supplemental Fig. 7).

The ultrastructure of tau inclusion was assessed by immuno-electron microscopy. These comprised abundant ~15.6-nm-diameter fibrils that are 1000–4000 nm in length labeled with anti-tau antibody 3026 (Fig. 5e, f), thereby confirming that tau inclusions were composed of the assembled tau filaments. Therefore, the tau inclusions in tau SPAM mice are biochemically and structurally similar to tau aggregates found in AD and other tauopathies.

### Pathogenic human tau recruits endogenous wild-type mouse tau into tau inclusions and promotes its hyperphosphorylation

**Fig. 6:**
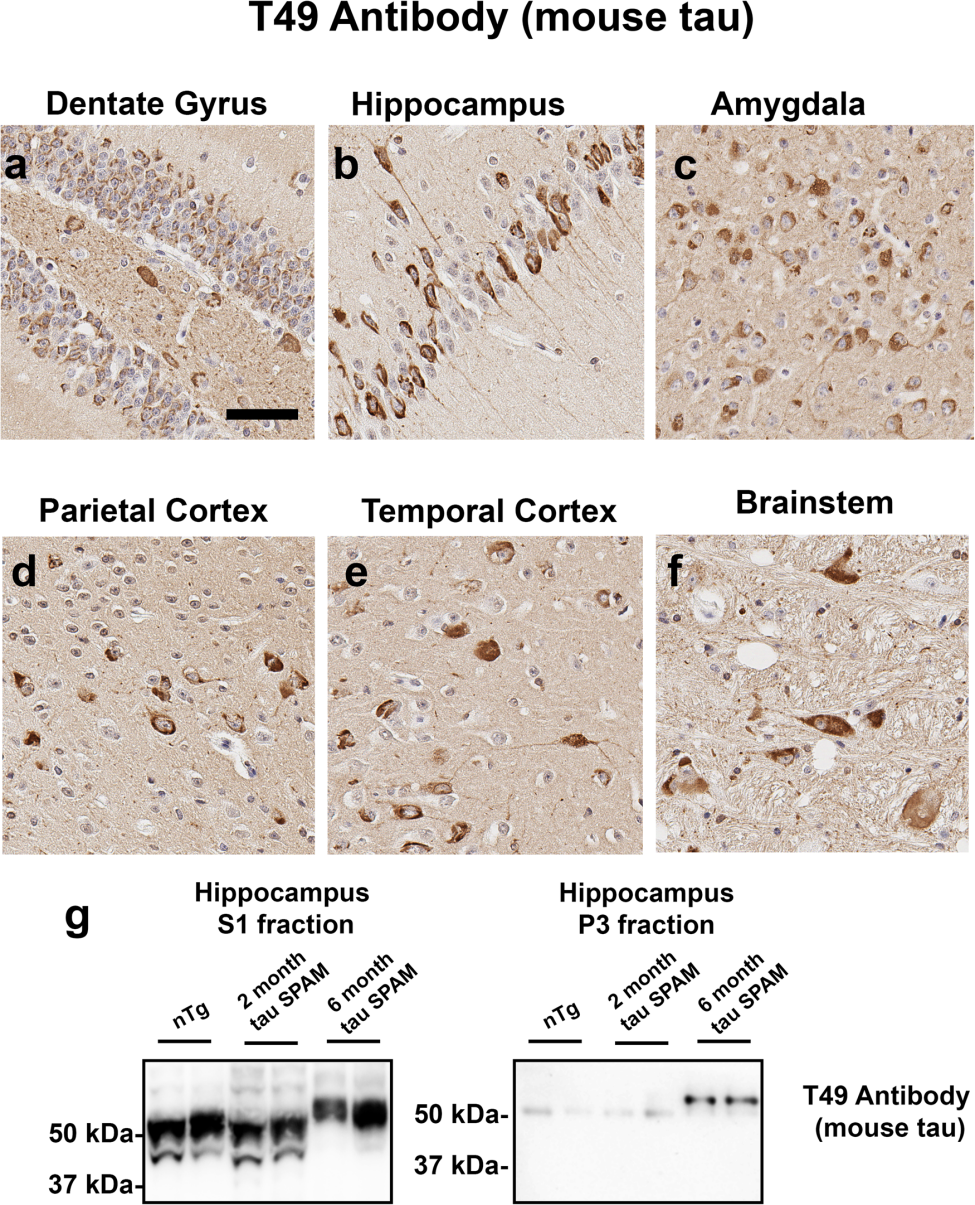
Mouse tau is recruited into neuronal inclusions of tau SPAM mice. IHC with antibody T49 specific for mouse tau strongly reveals NFT-like tau inclusions in (**a**) the dentate gyrus, (**b**) hippocampus, (**c**) amygdala, (**d**) parietal cortex, (**e**) temporal cortex, and (**f**) brainstem of 6-month-old tau SPAM mice. Scale bar = 100 μm. (**g**) Biochemical fraction followed by immunoblotting with T49 antibody reveals the temporal accumulation of aggregated, hyperphosphorylated (as reflected by the shift in reduced mobility) mouse tau in the hippocampus of tau SPAM mice. The relative molecular mass markers are shown on the left.

Since it has been suggested that mouse tau inhibits aggregation^45,46^, we investigated whether wild-type mouse tau is recruited into the tau aggregates of tau SPAM mice. T49 antibody specific for mouse tau^47^ was used to stain different brain regions of tau SPAM mice. Remarkably, mouse tau is rapidly incorporated into NFT-like tau inclusions in the cortex, hippocampus, brainstem, and other areas of significant tau pathology (Fig. 6a–f). Using the same T49 antibody, it was found that hyperphosphorylated mouse tau is specifically found in the detergent-insoluble P3 fraction in the hippocampus (Fig. 6g). Even in the soluble fraction, nearly all of the mouse tau was hyperphosphorylated at 6 months of age as reflected by the reduced mobility in molecular weight of the protein band on SDS-PAGE. This suggests that pathogenic human tau can recruit and sequester wild-type mouse tau into NFTs and that smaller tau aggregates of mouse and human tau are formed but remain in the S1 fraction (Figs. 3 and 6).

### Neuroinflammation is a late event that is only detectable in older tau SPAM mice

**Fig. 7:**
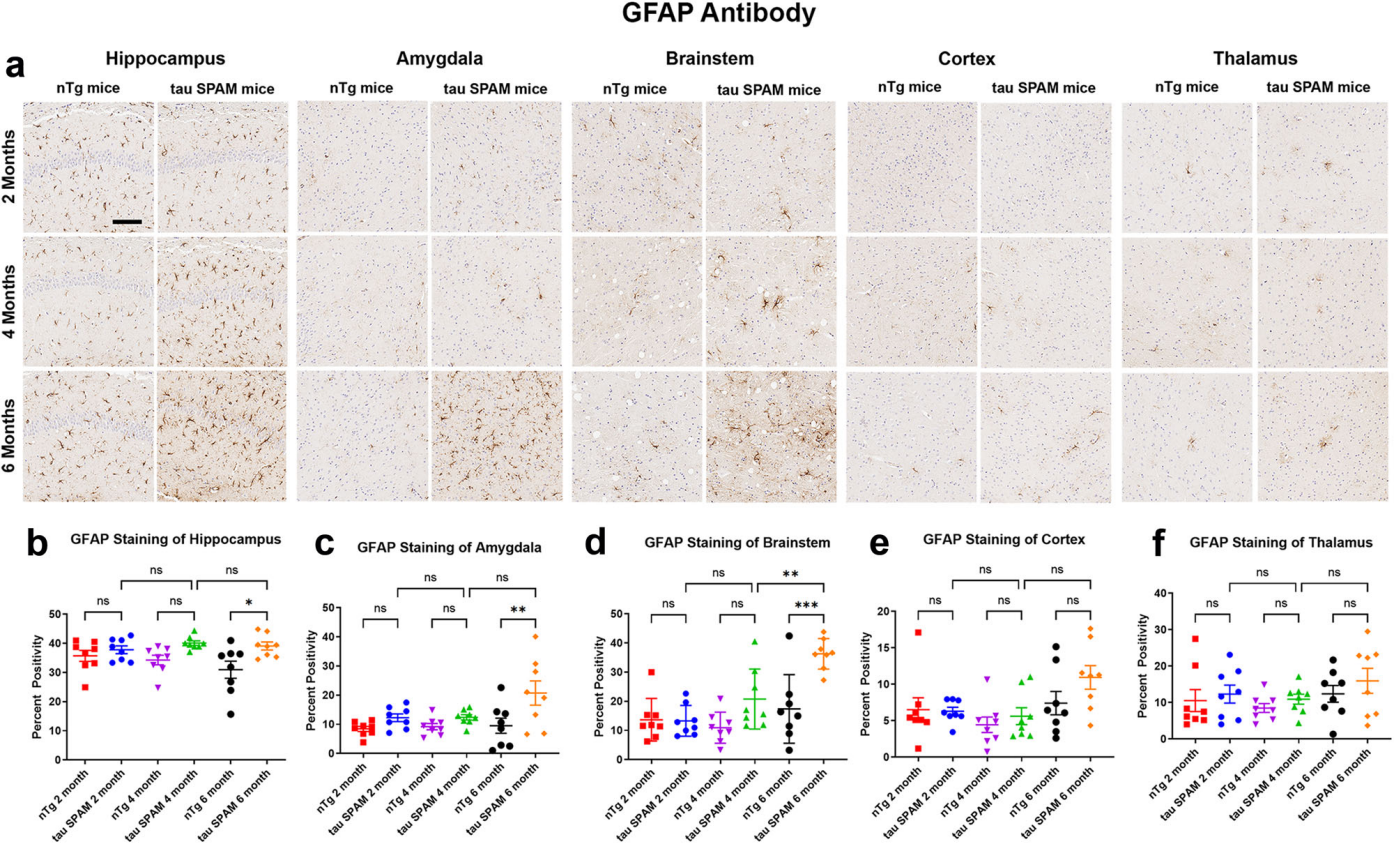
Astrogliosis is delayed compared with tau-inclusion formation in tau SPAM mice. **a** IHC with anti-GFAP antibody for reactive astrocytes was used to stain brain samples from tau SPAM mice and nTg mice at ages 2, 4, and 6 months. GFAP immunoreactivity was significantly increased in the (**b**) hippocampus [one-way ANOVA, F (5, 42) = 3.591, *p* = 0.0086], (**c**) amygdala [one-way ANOVA, F (5, 42) = 4.317, *p* = 0.0029], and (**d**) brainstem [one-way ANOVA, F (5, 42) = 10.84, *p* < 0.0001] at 6 months. (**e**) Cortex and (**f**) thalamus did not show significant increase in anti-GFAP immunoreactivity (*N* = 8, 4 M, 4 F for each time point). Scale bar = 100 μm. GFAP staining was quantified with Aperio algorithms and compared using one-way ANOVA with Dunnett’s test. Error bars represent standard errors of the mean. ***p < 0.001, **p < 0.01, *p < 0.05, ns not statistically significant.

Since neuroinflammation has been reported to be a common response to tau pathology^48^, we next sought to analyze the response of astrocytes and microglia to tau aggregates. While no major increase in GFAP-positive astrocytes was noted in 2- and 4-month-old mice, which already exhibit robust NFT-like pathology, an increase in GFAP staining was detected at 6 months in the areas with high density of tau inclusions, including hippocampus, amygdala, and brainstem, but not cortex and thalamus (Fig. 7). Similarly, increased CD11b-positive myeloid cells were evident but only in 6-month-old animals (Supplemental Fig. 8), and were noted in all examined brain regions. This suggests that NFT-like tau inclusions can persist in neurons for months before inducing significant neuroinflammatory responses.

### RNA-sequencing and transcriptomic analysis of tau SPAM mice reveals major pathway changes also implicated in sporadic AD

**Fig. 8:**
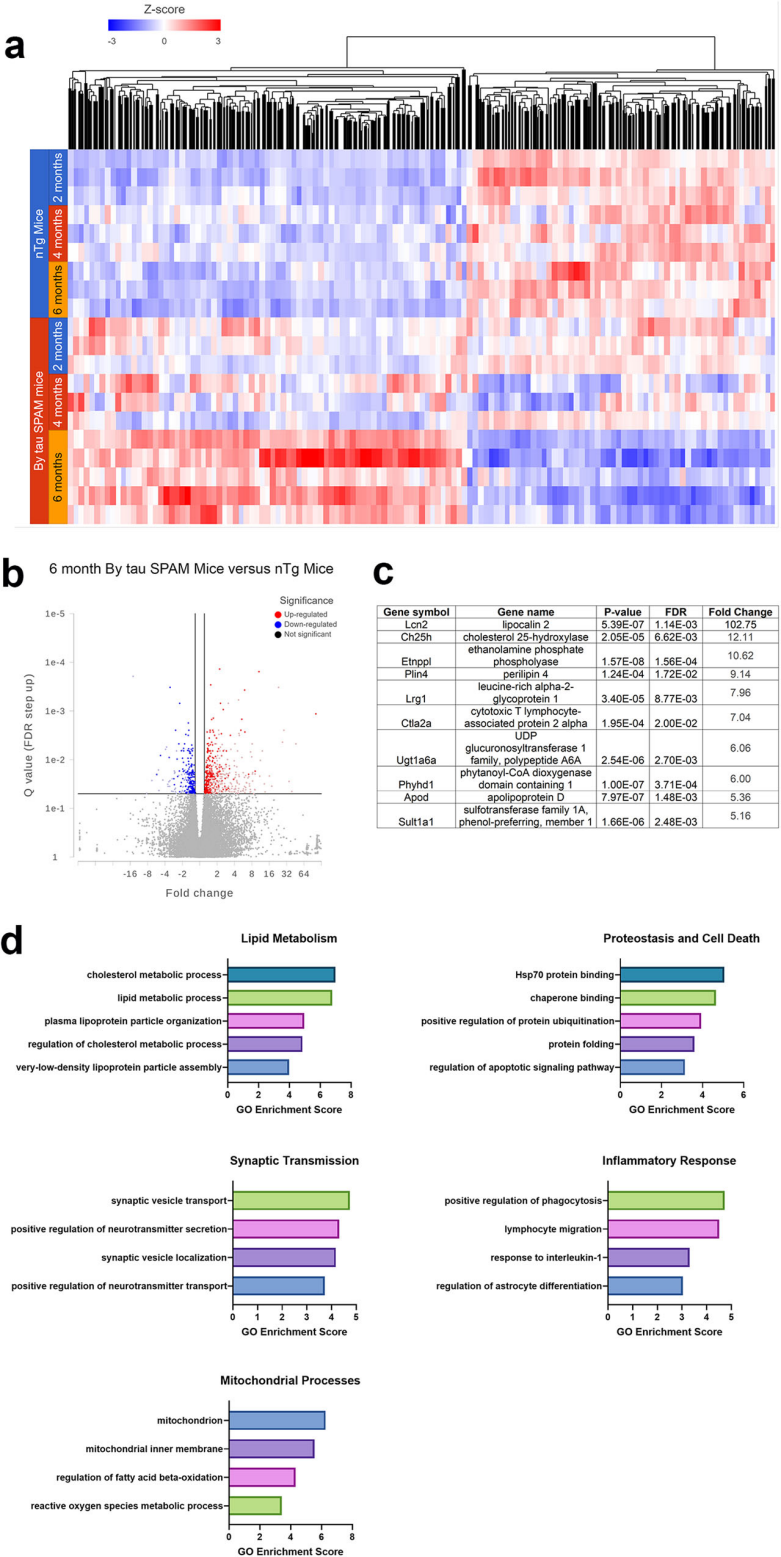
Major transcriptomic changes and pathway alterations are found primarily at 6 months with high tau inclusion density. **a** Heatmap shows hierarchical clustering of major gene expression changes between nTg and tau SPAM mice at 2, 4, and 6 months. **b** Volcano plot shows 473 differentially expressed genes between 6-month-old tau SPAM and nTg mice. **c** Top-ten significantly altered genes in tau SPAM mice based on fold change are listed in a table. **d** Major gene ontology enrichment and significant pathways are altered in tau SPAM mice, including lipid metabolism, proteostasis and cell death, synaptic transmission, inflammatory response, and mitochondrial processes.

RNA from cortical brain samples was isolated from 2-, 4-, and 6-month nTg and tau SPAM mice for RNA sequencing and transcriptomic analysis. Hierarchical clustering of significantly altered genes shows that major gene expression changes between nTg and tau SPAM mice occurred primarily at 6 months (Fig. 8a).

Gene-specific analysis for differential gene expression was performed between nTg and tau SPAM mice at 2, 4, and 6 months. There were no significantly altered genes between nTg and tau SPAM mice at 2 and 4 months with standard filtering criteria (described in Materials and methods). In 6-month-old tau SPAM mice, 473 out of 29,692 genes were found to be significantly altered and graphed as a volcano plot (Fig. 8b). The top-ten significantly altered genes by fold change are listed in table format (Fig. 8c). Many of these have been reported to be connected to AD and are associated with lipid metabolism (CH25H and APOD) or neuroinflammation (LCN2 and LRG1).

Gene ontology enrichment and pathway analysis of differentially regulated genes shows at least five major clusters: lipid metabolism, proteostasis and cell death, synaptic transmission, inflammatory response, and mitochondrial processes (Fig. 8d). Many of these pathways are known to be disrupted in AD and associated with neurodegeneration^49,50^, and are likely a response to significant tau toxicity.

### Intestinal dysfunction leads to impaired weight gain and sudden death

**Fig. 9:**
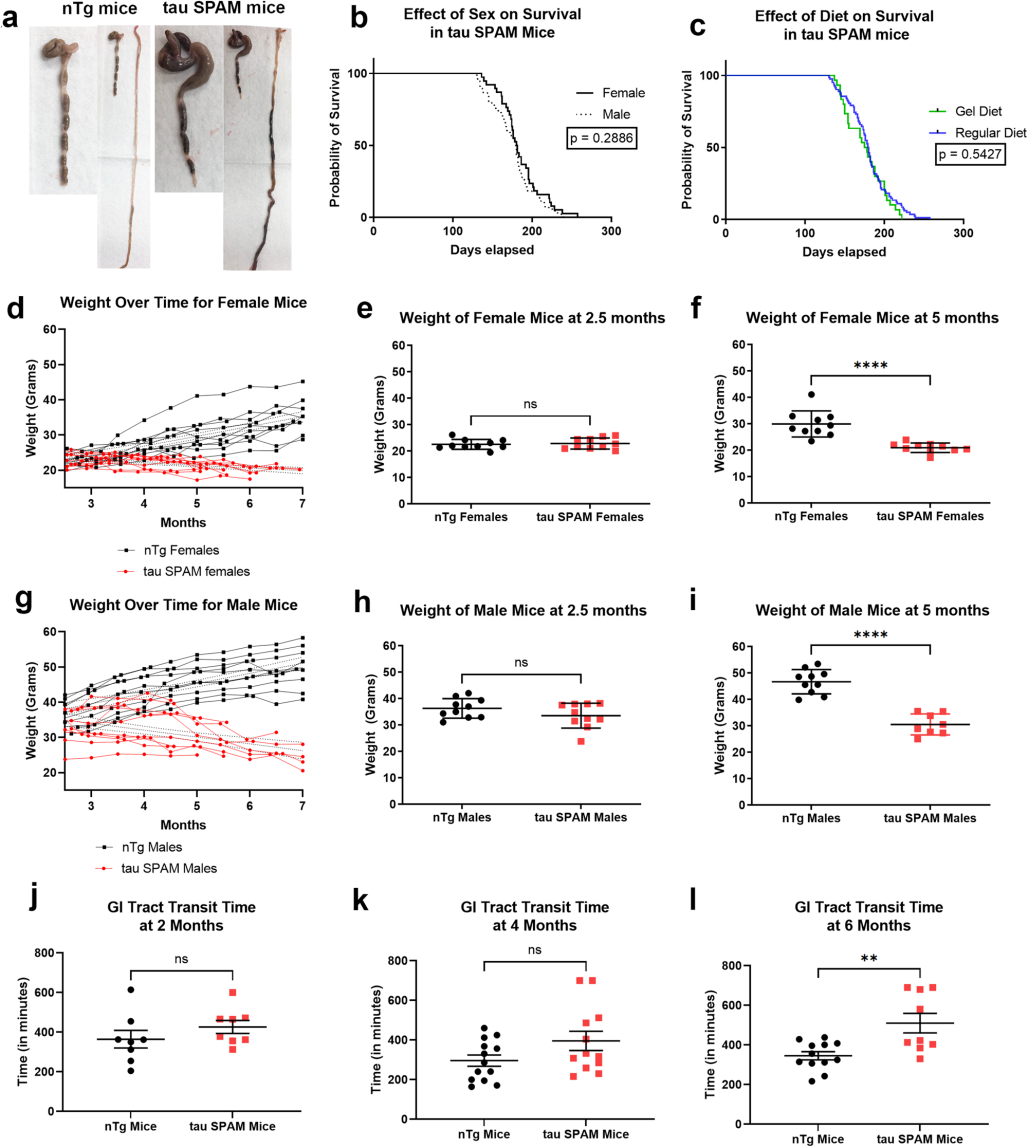
Weight-gain deficiency and sudden death with intestinal obstruction is a consistent phenotype at 6 months in both males and females of tau SPAM mice. **a** Images showing abdominal mass that develop intestinal obstruction around the distal colon in tau SPAM mice, but nTg mice do not have this phenotype. **b** Survival curve shows that the tau SPAM mice experience sudden death around 6 months (*N* = 38 females, and *n* = 44 males). No significant difference between survival for females (180.5 days) and males (179 days), *p* = 0.2886. **c** Severity of gut phenotype and mortality is not rescued by a gel diet. Tau SPAM mice were fed a gel-based low-fiber diet to try to alleviate intestinal symptoms (*N* = 30 mice). The survival curve was not altered by gel diet between mice on regular diet (179.5 days) and gel diet (176.5 days), *p* = 0.5427. **d**, **g** Weight was tracked over time from 2.5 months to 7 months in *N* = 20 (10 M, 10 F) for tau SPAM mice and nTg mice. Tau SPAM mice have significant lower weight after 3 months compared with nTg littermates. **e**, **h** At 2.5 months, tau SPAM mice do not have significantly different weights from nTg mice in both males (two-tailed t-test, *t* = 1.458, df = 18, *p* = 0.1620) and females (two-tailed *t*-test, *t* = 0.3474, df = 18, *p* = 0.7323). **f**, **i** At 5 months, tau SPAM mice experience significant lower weight in both males (two-tailed *t*-test, *t* = 7.866, df = 16, *p* < 0.0001) and females (two-tailed *t*-test, *t* = 5.399, df = 18, *p* < 0.0001). **j** At 2 months, gastrointestinal (GI) transit time is not statistically significant between tau SPAM and nTg mice (two-tailed *t*-test, *t* = 1.124, df = 14, *p* = 0.2800) for *N* = 8 in each group. ns not statistically significant. **k** At 4 months, GI transit time is not statistically significant between tau SPAM and nTg mice (two-tailed *t*-test, *t* = 1.796, df = 23, *p* = 0.0857) for *N* = 13 in nTg mice and *N* = 12 in tau SPAM mice. ns = not statistically significant. **l** At 6 months, GI transit time is significantly prolonged in tau SPAM mice compared with nTg mice (two-tailed *t*-test, *t* = 3.391, df = 19, *p* = 0.0031) for *N* = 12 in nTg mice and *N* = 9 in tau SPAM mice. Error bars represent standard errors of the mean. **p < 0.01.

At 6 months, most of the tau SPAM mice begin to experience spontaneous death. Around the endpoint, the mice were hunched, scruffy, and lethargic with signs of irritation. At necropsy, a significant number of mice had abdominal masses and intestinal obstruction of the distal colon (Fig. 9a). We tracked the death curve of males and females (*N* = 82 total) and found no major sex differences (Fig. 9b), *p* = 0.2886. Male mice died an average of 179 days (*N* = 44) and females died at an average of 180.5 days (*N* = 38). A modified gel-based diet was compared with regular diet to see if intestinal symptoms could be alleviated, which has been successful in TDP-43 mice^51^. However, gel diet did not significantly alter the survival curve in tau SPAM mice (Fig. 9c), *p* = 0.5427.

To analyze potential effects of the intestinal phenotype, the weight of tau SPAM mice and nTg littermates was tracked from 2.5 to 6 months every two weeks (Fig. 9d, g). While nTg littermate mice gain weight with age, SPAM mice fail to do so and their weight slightly declines after 3 months and until the terminal endpoint around 6 months. At 2.5 months, there were no significant changes in weight between nTg mice and tau SPAM mice in both males and females (Fig. 9e, h). By 5 months, tau SPAM mice displayed significant lower weight compared with nTg mice (Fig. 9h-i).

To identify the mechanism underlying the failure to gain weight in SPAM mice, we assessed the total gastrointestinal (GI) tract transit time by tracking food labeled with a dye. At 2 and 4 months, GI tract transit time was not statistically different between tau SPAM mice and nTg mice. At 6 months, GI tract transit time is significantly prolonged by ~50% in tau SPAM mice at 510.6 min (8.51 h), compared with 345.6 min (5.75 h) in nTg mice. This finding indicates age-dependent functional impairment of GI tract motility in tau SPAM mice that becomes pronounced at 6 months.

### Enteric neurons are susceptible to pathogenic phosphorylated tau resulting in neuronal death

**Fig. 10:**
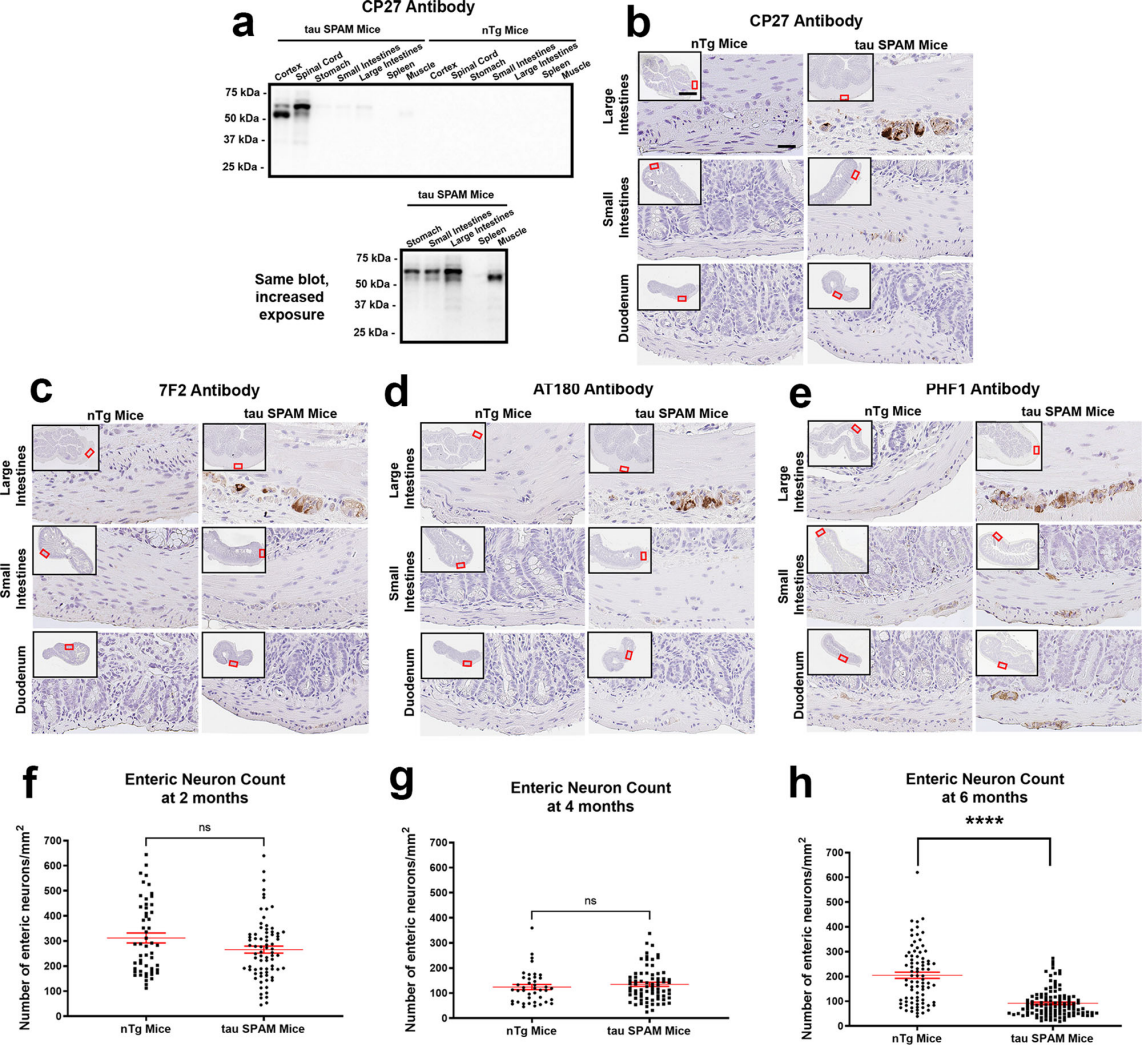
Enteric neurons are vulnerable to pathogenic tau and lead to significant enteric neuron loss in tau SPAM mice. **a** Immunoblot of lysates from different organs as indicated above each lane with antibody CP27 comparing expression in tau SPAM mice and nTg mice show that intestinal expression is very low relative to brain and spinal cord expression levels. 10 μg of protein was loaded per lane. Longer exposure of membrane (below) with only the lanes, including stomach, small intestines, large intestines, spleen, and muscle shows that most of the tau is shifted to relatively higher molecular mass band corresponding to hyperphosphorylated tau. The relative molecular mass markers are shown on the left. **b** CP27 immunostaining of duodenum, small intestines, and large intestines shows that human tau is more highly expressed in the large intestines. Scale bar = 50 μm for main image and 1 mm for inset. **c** Tau phosphorylation at Thr205 labeled with antibody 7F2 and (**d**) at Thr231 labeled with antibody AT180 is found primarily in large intestines of tau SPAM mice and not in nTg mice. **e** Increased PHF1 staining (against tau phosphorylated Ser396/Ser404) is found throughout the intestinal tract, particularly the large intestines of tau SPAM mice. **f** Random intestinal slides were sampled throughout the intestinal tract and stained with H&E for estimation of total enteric neurons. At 2 months, there was no statistically significant difference in enteric neuron density between tau SPAM mice and nTg mice (two-tailed *t*-test, *t* = 0.8162, df = 112, *p* = 0.4161) for *N* = 73 sections for tau SPAM mice and *N* = 41 sections for nTg mice. ns not statistically significant. **g** At 4 months, there was no statistically significant difference in enteric neuron density between tau SPAM mice and nTg mice (two-tailed *t*-test, *t* = 1.976, df = 127, *p* = 0.0503) for *N* = 76 sections in tau SPAM mice and *N* = 53 sections in nTg mice. ns not statistically significant. **h** At 6 months, tau SPAM mice had significant neuronal loss (~55% loss of neurons) compared with nTg mice (two-tailed *t*-test, *t* = 9.172, df = 195, *p* < 0.0001) for *N* = 115 sections in tau SPAM mice and *N* = 82 sections in nTg mice. Error bars represent standard errors of the mean. ****p < 0.0001.

Upon discovery of the intestinal phenotype, intestinal samples from duodenum, small intestines (jejunum and ileum), and large intestines were obtained to assess human tau expression relative to the brain and spinal cord. Overall, intestinal human tau expression was low compared with the CNS but detectable by immunoblotting (Fig. 10a). To better visualize the bands, the part of the immunoblot membrane with the intestinal tissues was cut out and overexposed (Fig. 10a, lower panel). This showed that within these tissues, large intestines had the highest expression levels and most of the tau found in the intestinal tract was hyperphosphorylated as upshifted to a higher molecular mass on SDS-PAGE.

Intestinal tissues were immunostained with CP27 antibody to determine the location of the cell types that expressed human tau. The only CP27-positive cells found were enteric neurons, primarily within the myenteric plexus. Intestinal tissue regions containing myenteric neurons were stained with several tau phosphorylation-specific antibodies (7F2, PHF1, and AT180) (Fig. 10b–e). In nTg mice, there was little to no staining for 7F2 and AT180, but there was some baseline-positive staining of PHF1 in some neurons. By comparison, tau SPAM mice had significantly higher staining in most neurons of each region, with significantly higher staining in the large intestines. While this staining does not appear to be NFT-like pathology, it does show increased somatodendritic accumulation of phosphorylated tau, comparable to pretangles. This was confirmed using all three phospho-antibodies, suggesting increased phosphorylation at multiple residues specifically within enteric neurons.

To determine potential enteric neuronal loss, random sections from duodenum, small, intestines, and large intestines were quantified by three different raters. At 2 and 4 months, there was no significant difference in enteric neuron density between nTg and tau SPAM mice (Fig. 10f, g). At older ages of 6 months, more than 50% of the neurons in enteric plexuses were lost in tau SPAM mice compared with nTg mice (Fig. 10h). The accumulation of hyperphosphorylated tau specifically within myenteric neurons likely contributes to neuronal dysfunction and death of enteric neurons followed by colonic obstruction and death of the mouse.

## Discussion

In the field of tauopathy, there is a compelling need for consistent and reproducible mouse models of tau pathology that can be used for rapid drug discovery, especially because over 300 therapies screened in murine models have been ineffective in human clinical trials^52^. Many tau models generated in the past have not been optimal for modeling progression of pathology and for rapid drug screening^8,53^. Popular mouse models such as rTg4510 model^54,55^ and PS19 mice^56^ express either the P301L or P301S tau mutation, respectively (summarized in Table 1). However, recent studies suggest that overexpression (12–13X levels) and disruption of developmental genes may account for the severe neuronal loss of tau pathology and brain atrophy in rTg4510 mice^57,58^. An attempt at recreating the rTg4510 mice is the rT2/T2 mouse line that develops tau inclusions by 5–7 months, but has 17X overexpression and has much slower onset of pathology^58^. An alternative model is the PS19 mice that are excellent for tau seeding experiments^59,60^; however, the inconsistency in the onset of tau pathology, ranging from 6 to 12 months, makes it difficult to assess tau-inclusion progression and potential therapeutic effects in that model^59,61^. PS19 mice also present major sex differences where females have a 5–6-month delay in pathology^62^, making it difficult to include both sexes in studies.

**Table 1:** Comparison of some commonly used transgenic mouse models of tauopathies to the new tau SPAM mice.

Model	Mutation with human tau isoform	Promoter	Expression relative to mouse tau	Tau pathology	Property
rTg4510 mice^54,55^	Inducible tTA, 0N4R P301L	CamKII	12–13X	Cortex, hippocampus at 4-5.5 months	Overexpression, disrupted developmental genes^57,58^
RT2/T2 mice^58^	Inducible tTA, 0N4R P301L	CamKII	17X	Cortex, hippocampus at 5–7 months	Overexpression, tTA disruption of several genes^58^
PS19 mice^56^	1N4R P301S	MoPrP	5X	Brainstem, hippocampus at 6-12 months	Inconsistent pathology^59,61^, paralysis^56^
tau SPAM mice	0N4R P301S/S320F	MoPrP	0.7X	Cortex, hippocampus, and other areas at 2-6 months	No sex difference, premature death from enteric neuronal loss

Therefore, our findings are impactful because this SPAM model of tauopathy provides major advantages that make it uniquely suitable for disease modeling and rapid drug screening, including: (1) accelerated and consistent tau pathology in disease-relevant brain areas, including the cortex and hippocampus. (2) Relatively lower transgenic tau expression levels (~0.7X-fold human tau compared with endogenous mouse tau). (3) Consistent and predictable death curve. (4) No major sex differences in tau burden or lethal phenotypic outcome.

The accelerated but progressive development of tau pathology from 2 to 6 months is particularly optimal for rapid drug screening. In the tau SPAM model of tauopathy, age-dependent progression of tau pathology in the cortex and hippocampus resembles progressive human pathology and develops significant inclusion density. Pathogenic tau is prone to hyperphosphorylation and leads to the accelerated formation of tau inclusions as early as 2 months in the cortex, hippocampus, and other major brain areas. These tau inclusions progressively increase with age from 2 to 6 months. At 6 months, tau pathology presents in nearly all areas of the neocortex, which closely resembles the endpoint tau burden in human patients. This was confirmed using multiple tau phosphorylation-specific antibodies, including 7F2, AT180, and PHF1 that readily detect human NFTs^33,34,37^. The accumulation of tau aggregates was also investigated by biochemical fractionation and immunoblotting: the temporal increase of Sarkosyl-insoluble tau aggregates is almost exclusively hyperphosphorylated similar to PHF-tau found in human tauopathies^63,64^. Unexpectedly, even tau in the detergent-soluble brain fractions becomes progressively hyperphosphorylated as observed by phospho-specific antibodies and the shift in reduced human tau mobility on SDS-PAGE.

The ultrastructure and amyloidogenic properties of the tau pathological inclusions in tau SPAM mice are similar to NFT found in human tauopathies as demonstrated by robust Thioflavin reactivity, silver staining, and immunolabeled EM, indicating that tau inclusions abundantly comprise tau fibrils^41–44^. Since only 4R tau was expressed, a limitation of this study is that mostly straight filaments are formed, while human tau inclusions consist of both straight and paired helical filaments.

Although overt neurodegeneration was not observed in the CNS, significant neuroinflammation (both microgliosis and astrogliosis) indicative of neuronal damage occurs at 6 months, which is significantly delayed compared with the appearance of tau inclusions. Previous studies have shown that uptake of tau seeds and aggregates by microglia induces the activation of NLRP3-ASC inflammasome pathway^65–67^. In our model, extensive tau pathology seems to sustain chronic neuroinflammation, which became more significant at older ages with progressive tau accumulation. Interestingly, we did not notice significant neuronal death in the cortex at 6 months, despite significant neuroinflammation, tau hyperphosphorylation, and tau aggregation. It is likely that neurons with tau pathology can survive for an extended period of time, which has also been confirmed in slice cultures where a subpopulation of tau inclusions can persist for months^68^. Likewise, other tau transgenic models show that neurons with inclusions can remain functional for a long time^69,70^ and it has been suggested that neurons can survive for decades with NFT before neuronal death in human patients^71^.

It was unexpected that wild-type mouse tau was extensively recruited into the tau inclusions of tau SPAM mice. Previously, it has been suggested that mouse tau inhibits tau aggregation and the absence of murine tau augments tau-inclusion formation^45,46,72^. In our new model, pathogenic human tau can directly recruit wild-type mouse tau into aggregates, resulting in concurrent hyperphosphorylation of mouse tau in both the soluble and detergent-insoluble biochemical fractions at 6 months but not at earlier ages. Pathogenic human tau likely sequesters mouse tau in both soluble oligomers (high-salt soluble fraction) and mature pathological inclusions (sarkosyl-insoluble fraction). This process likely requires time for both human and mouse tau to interact and is more significant at high densities of tau pathology.

PHF-tau isolated from AD brain has been shown to have prion-like properties, promoting the polymerization of wild-type tau and the formation of tau inclusions^73,74^. Additionally, injection of AD brain lysate containing hyperphosphorylated tau can induce aggregation of mouse tau in nTg mice^75,76^ and the prion-like spread of tau pathology can play a central role in tau-mediated progressive neurodegeneration. The extensive recruitment of mouse tau into the pathological inclusions of tau SPAM mice is consistent with a prion-like mechanism and may also partially explain the accelerated timeline of tau pathology in these mice, compared with other tau mouse models. Tau with a mutation at only Pro301 (e.g., P301L, P301S, and P301T) is not particularly prone to intrinsically aggregate without induction by prion-like seeding as shown by both in vitro and in cellular aggregation studies^11,77,78^. Furthermore, tau aggregates from patients with Pro301 mutations have not been reported to recruit wild-type human tau, suggesting that the SPAM tau has unique strain-dependent properties^79,80^. The property of SPAM tau to recruit wild-type mouse tau indicates that the structural and folding properties of the SPAM tau prion-like strain are more congruent with wild-type tau. Compared with tau transgenic mice that only express P301L or P301S, tau SPAM mice may more closely model the properties of human tau that occur in brains from subjects with AD.

To better understand the overall response to tau pathology, RNA-sequencing and transcriptomic analysis was performed on cortex samples for nTg and SPAM mice at different ages of 2, 4, and 6 months. The cortex was deemed to be a good target for analysis since tau-inclusion density progressively increased with age. Surprisingly, no differentially expressed genes were found at 2 and 4 months, but at least 473 genes were significantly altered at 6 months. This further confirms that some neurons can tolerate tau aggregates for at least some time until they are unable to compensate for higher tau density. At 6 months, many of the same pathways altered in human AD patients are altered in SPAM mice, including lipid metabolism, proteostasis and cell death, synaptic transmission, neuroinflammation, and mitochondrial processes. In particular, neuroinflammation and lipid metabolism alterations are found in sporadic AD and neurodegeneration^49,50^, which is further supported by the increased astrogliosis and microgliosis found at 6 months. Among the list of top-ten genes with the most significant changes, lipocalin-2 (LCN) was markedly elevated only at 6 months. LCN2 is released by reactive astrocytes as a part of the neuroinflammatory response and can lead to synaptic impairment and neuronal death^81,82^. Clinically, LCN2 has been identified as a plasma biomarker of preclinical AD and in patients with mild cognitive impairment^83,84^. Other top-10 genes are involved in lipid metabolism: cholesterol 25-hydroxylase (CH25H) is a major AD risk gene found on Chromosome 10q^85^ and apolipoprotein D (APOD) is an AD marker significantly upregulated in the brain^86,87^. Genes such as Phyhd1 are associated with the molecular signature of NFT progression^88^. Overall, the transcriptomic and pathway changes in tau SPAM mice closely model those of sporadic AD progression. Although some changes may be due to general sickness during the terminal endpoint, most of the transcriptomic changes were CNS-specific and are likely a result of toxic tau aggregates.

In addition to affecting the CNS, tau SPAM mice also develop intestinal obstruction and dysfunction from the expression of tau in enteric neurons. Intestinal dysfunction has also been observed in other transgenic mice expressing SCA7 or TDP-43 mutations using the MoPrP promoter^89,90^. Different intestinal disorders including colon cancer are associated with AD, and intestinal symptoms such as constipation are common in AD^91,92^. Chronic constipation from aging has also been associated with enteric neurodegeneration^25,26^ with evidence of enteric neuron loss in dementia^27^. Additionally, inflammatory bowel disease such as Crohn’s disease has increased tau expression in enteric neurons^93^. However, there is a gap in the literature on tau since intestinal samples from AD and FTD patients are not commonly collected^23^. In one study, more AD and FTD cases have pT231 staining in the sigmoid colon compared with nondemented control cases^29^, which was confirmed by increased Thr231 phosphorylation in tau SPAM mice.

Although it is difficult to compare the absolute cellular expression levels of human tau between gut and brain, as the density of neurons is much lower in the intestine compared with the CNS, the overall expression of SPAM tau in the intestines was less than in the brain. This is suggestive of the notion that enteric neurons may be more susceptible to tau-induced neurotoxicity as the accumulation of hyperphosphorylated tau resulted in a significant loss (~55%) of the neurons of the myenteric plexus at 6 months, but not at early ages of 2 and 4 months. Functionally, enteric neuronal loss results in significantly delayed colonic transit time at 6 months. This progressive neuronal decline contributes to a lack of weight gain after 2–3 months likely due to decreased appetite to compensate for bloating and fecal retention. As far as we are aware, this is the first tau model, which shows that pathogenic tau can be hyperphosphorylated in myenteric neurons and lead to enteric neuronal death and functional impairment. As a model used for rapid drug screening, it will be advantageous to test the effects of lead compounds on the effects of pathogenic tau in both the gut and brain.

In conclusion, the tau SPAM mice develop robust and widespread neuronal amyloidogenic tau inclusions that closely model human NFT. The accelerated tau pathology appears as early as 2 months and reaches high tau-inclusion density by 4–6 months. Additionally, these mice have a robust enteric neurodegeneration phenotype, consistent mortality curve, low transgene expression levels, and no major sex differences ideal for rapid screening of tau-targeting therapies.

## Materials and methods

### Creation of Tau transgenic mice and mouse husbandry

All mouse experimental procedures and husbandry were approved by the University of Florida Institutional Animal Care and Use Committee. 0N4R human isoform cDNA with the S320F and P301S mutations was cloned into the MoPrP.Xho vector that drives expression in nearly all neurons of the central nervous system by the mouse prion promoter^10^. This construct was injected into zygote-fertilized eggs of C3H/BL6 mice by the University of Pennsylvania Transgenic & Chimeric Mouse Facility (Director Dr. Jean Richa), which is supported by the Institute for Diabetes, Obesity, and Metabolism, the Center for Molecular Studies in Digestive and Liver Diseases, and the Abramson Cancer Center.

Five founder lines of mice were transferred from the University of Pennsylvania and bred in-house at the University of Florida under a 12-hour light/dark cycle with unlimited access to food and water. Four lines demonstrated germline-stable transmission with expression of human tau. The highest expressing line, tau SPAM mice, was analyzed for this study. A cohort of these mice was maintained on Diet Gel 76A (ClearH_2_O, Portland, ME) starting at 4–4.5 months of age.

The transgene-integration site for this line was determined by targeted locus amplification (TLA) provided as a service by Cergentis (Utrecht, Netherlands). Genome sequencing identified that the tau transgene was inserted at mouse chr7:118,151,846–118,283,375 and replaced three genes localized within the chromosome region (*Smg1*, *4930583K01 Rik*, and *Gm34999*). Both *4930583K01 Rik* and *Gm34999* are noncoding genes that are likely not expressed. The only coding gene is *SMG1*: homozygous knockout mouse for *SMG1* is embryonically lethal, but the heterozygous *SMG1* knockout develops normally without noticeable effects on CNS or intestinal tissue^94^.

### Antibodies

Total tau rabbit polyclonal antibody 3026 was previously described^37^. CP27 is a mouse monoclonal antibody specific for human tau^95,96^, and T49 is a mouse monoclonal antibody specific for mouse tau^47^ (Thermo Fisher Scientific, Waltham, MA). The following phosphorylation-specific tau mouse monoclonal antibodies were also used: 7F2 specific for phosphorylation at Thr205^37^, AT180 specific for phosphorylation at Thr231 (Thermo Fisher Scientific, Waltham, MA)^34^, and PHF1 specific for phosphorylation at S396 and S404^33^. CP27, PHF1 and CP13 antibodies were generous gifts from the late Dr. Peter Davies.

Other antibodies used were rabbit polyclonal antibody specific for anti-glial fibrillary acid protein (GFAP) (Dako/Agilent Technologies, Santa Clara, CA), rabbit monoclonal antibody specific for CD11b (clone EPR1344, Abcam, Cambridge, MA), mouse monoclonal antibody raised against ubiquitin (clone Ubi-1, EMD Millipore, Burlington, MA), rabbit polyclonal antibody specific for p62/SQSTM1 (Proteintech, Rosemont, IL), mouse monoclonal antibody specific for actin (clone C4, Fisher Scientific), and mouse monoclonal antibody specific for glyceraldehyde-3-phosphate dehydrogenase (GAPDH, clone GA1R, Fisher Scientific).

### Western blotting

Different organs as indicated in the figure legends, including brain and spinal cord, were extracted by sonication in 50 mM Tris, pH 7.5/2% SDS and heated to 100 °C for 10 min. Protein concentration was determined using the bicinchoninic acid (BCA) assay (Thermo Fisher Scientific) and bovine serum albumin as the standard. Equal amounts of protein were loaded on 10% SDS-polyacrylamide gels, separated by SDS-PAGE, and electrophoretically transferred onto nitrocellulose membranes. Membranes were blocked in Tris-buffered saline (TBS) with 5% powdered milk for 1 h at room temperature and incubated in primary antibodies overnight at 4 °C. After washes, the membranes were incubated for an hour in anti-rabbit or anti-mouse secondary antibodies conjugated to horseradish peroxidase (Jackson ImmunoResearch, West Grove, PA). For visualization, the membranes were exposed with Western Lightning Plus ECL reagents (PerkinElmer Life Sciences, Waltham, MA) and imaged by chemiluminescence (PXi, Syngene, Frederick, MD). Each lane was quantified based on densitometric analysis with ImageJ software. Statistical tests were calculated on GraphPad Prism for one-way or two-way analysis of variance (ANOVA) with post hoc analysis by Dunnett’s test.

### Tau dephosphorylation assay

Brain and spinal cord lysates from tau SPAM mice and nTg mice were homogenized by sonication in 4 mL/g of buffer (0.75 M NaCl, 100 mM Tris, 1 mM EDTA, 0.5 mM MgSO4, and 2 mM DTT) with a mix of protease inhibitors (see below). The homogenates were centrifuged at 21,000 × *g* for 5 min. The supernatant was collected and heated at 100 °C for 5 min followed by centrifugation at 21,000 × *g* for 5 min. The supernatant was then diluted 1:10 in 50 mM HEPES, pH 7.5, 100 mM NaCl, 2 mM DTT, 0.01% Brij-35, and 1 mM MnCl_2_. 50 uL of each sample was divided into control and reaction groups. 2 uL (400 units/ul) of Lambda Protein Phosphatase (New England BioLabs, Ipswich, MA) was added to the reaction group. All samples were then incubated at 37 °C for 3 h. SDS was added to a final concentration of 2% to each tube, and the samples were heated at 95 °C for 10 min. The samples were frozen at −80 °C and thawed and used for western blot after adding sample buffer.

### Sequential brain fractionation for isolation of protein aggregates

Sequential detergent extraction was used to isolate detergent-insoluble tau aggregates in multiple fractions. Frontal cortex, hippocampus, and spinal cord were isolated from transgenic tau SPAM mice and nontransgenic (nTg) littermates at ages of 2, 4, and 6 months. These samples were homogenized by sonication in 3 mL per gram of high-salt (HS) buffer (50 mM Tris-HCl, pH 7.5, 0.75 M NaCl, and 2 mM EDTA) containing a cocktail of proteinase inhibitors (1 mM phenylmethylsulfonyl fluoride and 1 μg/ml each of pepstatin, leupeptin, *N*-tosyl-l-phenylalanyl chloromethyl ketone, *N*-tosyl-lysine chloromethyl ketone, and soybean trypsin inhibitor) and centrifuged at 100,000 × g at 4 ^o^C to separate into a soluble HS fraction (S1) and pellet, as previously described^37,97^. The pellet was redissolved in 2 mL per gram of HS buffer with 1% Triton X-100 and centrifuged again to separate into a Triton-soluble fraction (S2) and pellet. As a wash step, the pellet was solubilized in 2 mL per gram of HS buffer with 1% Triton X-100 and 1 M sucrose to remove myelin. For the final extraction step, the pellet was redissolved in 1 mL per gram of HS buffer with 1% sarkosyl. This was centrifuged and separated into a sarkosyl-soluble fraction (S3) and the pellet (P1), which was solubilized in 0.5 mL per gram of SDS-urea buffer (2% SDS, 4 M urea, and 25 mM Tris-HCl, pH 7.6).

### Immunohistochemistry and quantitative analysis

Mouse tissue from brain, spinal cord, and intestines was fixed in either 70% ethanol/150 mM NaCl or formalin and embedded in paraffin before staining. Slides were rehydrated in xylene and ethanol solutions (100%, 90%, and 70%). For antigen retrieval, slides were heated in a steam bath for 30 min in water with 0.05% Tween-20. Slides were incubated in PBS with 1.5% hydrogen peroxide and 0.005% Triton X-100 to quench endogenous peroxidase. After washes, slides were blocked in 2% FBS/0.1 M Tris, pH 7.6, and were incubated in primary antibody overnight at 4 °C. The next day, slides were incubated with biotinylated anti-mouse secondary antibody or biotinylated anti-rabbit secondary antibody (Vector Laboratories, Burlingame, CA) for 1 h and streptavidin-conjugated HRP (Vectastain ABC kit from Vector Laboratories, Burlingame, CA) for 1 h. Slides were developed in 3,3′-diaminobenzidine (DAB kit; KPL, Gaithersburg, MD) and counterstained with Mayer’s hematoxylin (Sigma-Aldrich, St. Louis, MO). The slides were dehydrated in ethanol solutions (70%, 90%, and 100%) followed by xylene and coverslipped with Cytoseal 60.

Quantitative analysis of IHC staining was performed using Imagescope software with Aperio algorithms. For counting of neuron density, NeuN antibody (Sigma-Aldrich, St. Louis, MO) was used to stain different sections and quantified using the nuclear counting algorithm. Additionally, this nuclear counting algorithm was modified to quantitatively count the total number of tau inclusions in different brain areas within a fixed-size square for different tau antibodies. For the hippocampus with higher density of neurons, the color-deconvolution algorithm was used to measure the percentage of positive staining around the outline of the hippocampal areas. CD11b and GFAP staining was also quantified based on positive pixel count.

### Silver and Thioflavin-S staining

A modified Campbell–Switzer silver stain protocol was used to label silver-positive inclusions^98^. The samples were rehydrated and incubated in 2% ammonium hydroxide for 15−20 min. The slides were transferred to a silver-pyride-carbonate solution for 40 min without light. After rinsing in 1% citric acid and acetate buffer (pH 5.0), the slides were developed with silver nitrate and aldehyde.

For Thioflavin-S staining, paraffin-embedded tissue sections were rehydrated and incubated in 0.25% potassium permanganate and 1% potassium metabisulfite/1% oxalic acid for 5 min each. Slides were submerged in 0.02% Thioflavin S for 10 min and then rinsed in water and 70% ethanol. Stained slides were mounted on aqueous fluoromount and images captured with an Olympus BX51 fluorescence microscope (Olympus, Center Valley, PA).

### Immuno-electron microscopy

Hippocampal and cortical samples fixed in 4% paraformaldehyde/0.25% glutaraldehyde in PBS overnight, followed by washes with PBS, and cut with a vibratome. After blocking with 5% FBS/1% BSA in PBS, the brain sections were incubated in 0.1% BSA/PBS with primary antibody 3026 specific for total tau overnight. After washes, anti-rabbit HRP secondary antibody and DAB was used to visualize tau inclusions. The sections were further stained with silver intensification^99,100^, dehydrated in ethanol, and embedded in Embed 812 resin blocks (Electron Microscopy Sciences, Hatfield, PA). Samples were loaded onto grids and images were captured with a Hitachi 7600 transmission electron microscope with an AMT digital camera.

### RNA sequencing and analysis

Brain cortex samples were taken from 2-, 4-, and 6-month-old nTg mice and tau SPAM mice. RNA extraction and RNA sequencing were performed as a service by BGI Genomics (Cambridge, MA) using the DNBSEQ™ Technology Platform. Raw reads were verified to have a Phred quality score >35 indicating high accuracy of reads. Gene-specific analysis for differential expression and statistical analysis were performed in Partek^®^ Flow^®^ software. Filtering criteria for differential genes were *p*-value <0.05, false-discovery rate with *q*-value <0.05, and total reads >10. Differentially expressed genes were analyzed for gene ontology enrichment and pathway changes in Partek^®^ Flow^®^. Significantly altered genes and pathways (*p*-value <0.05) were clustered and graphed based on enrichment score and relevance.

### Hematoxylin and eosin staining and quantitative counting of enteric neurons

Intestinal samples were obtained from duodenum, small intestines, and large intestines, and fixed in formalin for 1 day and transferred to 70% ethanol/150 mM NaCl. For hematoxylin and eosin staining (H&E), slides were rehydrated in xylene and ethanol series before counterstaining with Mayer’s hematoxylin. After washing in water, the slides were placed in 95% EtOH for several minutes before staining with 99.5% eosin Y with 0.5% acetic acid. The slides were dehydrated in 100% ethanol and xylene before coverslipping with Cytoseal 60.

Random cross sections were sampled from duodenum, small intestines, and large intestines. A blinded observer took 10–20 snapshots of each region for quantitative counting. Three different blinded scorers (BMB, SP, and YX) counted the total number of enteric neurons in each snapshot for every region. The average of these counts was graphed and compared using paired t-tests at different ages of 2, 4, and 6 months. Four outliers were removed from the dataset based on Grubb’s test. To further validate neuronal counts, the interrater coefficient (ICC) was calculated to be 0.951 (95% confidence interval of 0.943–0.0.958), which suggests that the counts are highly correlative between different raters.

### Gastrointestinal tract transit-time assay

Similar to previous studies^101^, both tau SPAM mice and nTg mice were treated by oral gavage with 0.3 ml of 6% (w/v) carmine red dye in 0.5% methyl cellulose after fasting for at least 1 h. After treatment, mice were checked every 15–30 min, until the first appearance of red dye in fecal matter. The final times were recorded (up to 700 min) and graphed for analysis.

### Statistics and reproducibility

Statistical analysis and graphs were made using Graphpad Prism 9 software for either paired *t*-tests or ANOVAs. The results are reported with statistical tests, *p*-values, and F-tests for distribution for each figure in the figure legends. Error bars on graphs represent standard errors of the mean. Sample sizes are reported as separate mice (n) with equal males and females for each individual experiment.

### Reporting summary

Further information on research design is available in the Nature Research Reporting Summary linked to this article.

## Supplementary information

Supplemental Information

Description of Additional Supplementary Files

Supplementary Data 1

Reporting Summary

## Data Availability

The raw and processed data for RNA-sequencing analysis have been deposited in NCBI’s Gene Expression Omnibus and can be accessed using the GEO Series accession number GSE198667. The source data underlying Figs. 4, 7, 8, 9 and 10 are provided as Supplemental Data 1. Uncropped blots used in Figs. 1, 3, 6 and 10 are included in Supplemental Information. Any additional relevant data that support the findings of this study are available from the corresponding author (BIG) upon reasonable request.

## Material availability

Unique biological materials such as antibodies and transgenic mice are available upon request according to the University of Florida MTA guidelines.

## References

[1] Qiu C, Kivipelto M, von Strauss E (2009). Epidemiology of Alzheimer’s disease: occurrence, determinants, and strategies toward intervention. Dialogues Clin. Neurosci..

[2] Jack CR (2018). NIA-AA research framework: toward a biological definition of Alzheimer’s disease. Alzheimers Dement..

[3] Hutton M (1998). Association of missense and 5′-splice-site mutations in tau with the inherited dementia FTDP-17. Nature.

[4] Wang Y, Mandelkow E (2016). Tau in physiology and pathology. Nat. Rev. Neurosci..

[5] Galimberti D, Scarpini E (2012). Genetics of frontotemporal lobar degeneration. Front. Neurol..

[6] Kametani F, Hasegawa M (2018). Reconsideration of Amyloid hypothesis and Tau hypothesis in Alzheimer’s disease. Front. Neurosci..

[7] Myers, A. & McGonigle, P. Overview of transgenic mouse models for Alzheimer’s disease. *Curr. Protoc. Neurosci*. **89**, e81 (2019). 10.1002/cpns.81 31532917

[8] Joel Z (2018). Improving mouse models for dementia. Are all the effects in tau mouse models due to overexpression?. Cold Spring Harb. Symp. Quant. Biol..

[9] Jankowsky JL, Zheng H (2017). Practical considerations for choosing a mouse model of Alzheimer’s disease. Mol. Neurodegener..

[10] Borchelt DR (1996). A vector for expressing foreign genes in the brains and hearts of transgenic mice. Genet. Anal..

[11] Strang KH (2018). Distinct differences in prion-like seeding and aggregation between Tau protein variants provide mechanistic insights into tauopathies. J. Biol. Chem..

[12] Croft CL (2019). rAAV-based brain slice culture models of Alzheimer’s and Parkinson’s disease inclusion pathologies. J. Exp. Med..

[13] Koller EJ (2019). Combining P301L and S320F tau variants produces a novel accelerated model of tauopathy. Hum. Mol. Genet..

[14] Xia Y (2019). Impaired tau-microtubule interactions are prevalent among pathogenic tau variants arising from missense mutations. J. Biol. Chem..

[15] Bugiani O (1999). Frontotemporal dementia and corticobasal degeneration in a family with a P301S mutation in tau. J. Neuropathol. Exp. Neurol..

[16] Baba Y (2007). Clinical and genetic features of families with frontotemporal dementia and parkinsonism linked to chromosome 17 with a P301S tau mutation. J. Neural Transm..

[17] Rosso SM (2002). A novel tau mutation, S320F, causes a tauopathy with inclusions similar to those in Pick’s disease. Ann. Neurol..

[18] Melis V (2015). Different pathways of molecular pathophysiology underlie cognitive and motor tauopathy phenotypes in transgenic models for Alzheimer’s disease and frontotemporal lobar degeneration. Cell. Mol. Life Sci..

[19] Schindowski K (2006). Alzheimer’s disease-like tau neuropathology leads to memory deficits and loss of functional synapses in a novel mutated tau transgenic mouse without any motor deficits. Am. J. Pathol..

[20] Rosenmann H (2008). A novel transgenic mouse expressing double mutant tau driven by its natural promoter exhibits tauopathy characteristics. Exp. Neurol..

[21] Oddo S (2003). Triple-transgenic model of Alzheimer’s disease with plaques and tangles: intracellular Abeta and synaptic dysfunction. Neuron.

[22] Oakley H (2006). Intraneuronal beta-amyloid aggregates, neurodegeneration, and neuron loss in transgenic mice with five familial Alzheimer’s disease mutations: potential factors in amyloid plaque formation. J. Neurosci..

[23] Niesler, B., Kuerten, S., Demir, I. E. & Schäfer, K. H. Disorders of the enteric nervous system — a holistic view. *Nat. Rev. Gastroenterol. Hepatol.* (2021). 10.1038/s41575-020-00385-2 10.1038/s41575-020-00385-2 33514916

[24] Chalazonitis A, Rao M (2018). Enteric nervous system manifestations of neurodegenerative disease. Brain Res..

[25] Camilleri M, Cowen T, Koch TR (2008). Enteric neurodegeneration in ageing. Neurogastroenterol. Motil..

[26] Wiskur B, Greenwood-Van Meerveld B (2010). The aging colon: the role of enteric neurodegeneration in constipation. Curr. Gastroenterol. Rep..

[27] Bassotti G (2007). Apoptotic phenomena are not a major cause of enteric neuronal loss in constipated patients with dementia. Neuropathology.

[28] Tam PKH (1990). An immunohistological study of the human enteric nervous system with microtubule-associated proteins. Gastroenterology.

[29] Dugger BN (2019). Tau immunoreactivity in peripheral tissues of human aging and select tauopathies. Neurosci. Lett..

[30] Gozal YM (2009). Proteomics analysis reveals novel components in the detergent-insoluble subproteome in Alzheimer’s disease. J. Proteome Res..

[31] Diner, I., Nguyen, T. & Seyfried, N. T. Enrichment of detergent-insoluble protein aggregates from human postmortem brain. *J. Vis. Exp*., 10.3791/55835 (2017). 10.3791/55835 PMC5755167 29155708

[32] Goedert M, Jakes R, Vanmechelen E (1995). Monoclonal antibody AT8 recognises tau protein phosphorylated at both serine 202 and threonine 205. Neurosci. Lett..

[33] Otvos L (1994). Monoclonal antibody PHF-1 recognizes tau protein phosphorylated at serine residues 396 and 404. J. Neurosci. Res..

[34] Goedert M (1994). Epitope mapping of monoclonal antibodies to the paired helical filaments of Alzheimer’s disease: identification of phosphorylation sites in tau protein. Biochem. J..

[35] Wesseling H (2020). Tau PTM profiles identify patient heterogeneity and stages of Alzheimer’s disease. Cell.

[36] Xia Y, Prokop S, Giasson BI (2021). “Don’t Phos Over Tau”: recent developments in clinical biomarkers and therapies targeting tau phosphorylation in Alzheimer’s disease and other tauopathies. Mol. Neurodegener..

[37] Strang KH (2017). Generation and characterization of new monoclonal antibodies targeting the PHF1 and AT8 epitopes on human tau. Acta Neuropathol. Commun..

[38] Babu JR, Geetha T, Wooten MW (2005). Sequestosome 1/p62 shuttles polyubiquitinated tau for proteasomal degradation. J. Neurochem..

[39] Perry G, Friedman R, Shaw G, Chau V (1987). Ubiquitin is detected in neurofibrillary tangles and senile plaque neurites of Alzheimer disease brains. Proc. Natl Acad. Sci. USA.

[40] Kuusisto E, Salminen A, Alafuzoff I (2002). Early accumulation of p62 in neurofibrillary tangles in Alzheimer’s disease: possible role in tangle formation. Neuropathol. Appl. Neurobiol..

[41] Guntern R, Bouras C, Hof PR, Vallet PG (1992). An improved thioflavine S method for staining neurofibrillary tangles and senile plaques in Alzheimer’s disease. Experientia.

[42] Kelényi G (1967). Thioflavin S fluorescent and congo red anisotropic stainings in the histologic demonstration of amyloid. Acta Neuropathol..

[43] Iqbal K, Braak E, Braak H, Zaidi T, Grundke-Iqbal I (1991). A silver impregnation method for labeling both Alzheimer paired helical filaments and their polypeptides separated by sodium dodecyl sulfate-polyacrylamide gel electrophoresis. Neurobiol. Aging.

[44] Iqbal K, Braak H, Braak E, Grundke-Iqbal I (1993). Silver labeling of alzheimer neurofibrillary changes and brain β-amyloid. J. Histotechnol..

[45] Ando K (2010). Deletion of murine tau gene increases tau aggregation in a human mutant tau transgenic mouse model. Biochem. Soc. Trans..

[46] Ando K (2011). Accelerated human mutant tau aggregation by knocking out murine tau in a transgenic mouse model. Am. J. Pathol..

[47] Ishihara T (2001). Attenuated neurodegenerative disease phenotype in tau transgenic mouse lacking neurofilaments. J. Neurosci..

[48] Marttinen M (2018). Molecular mechanisms of synaptotoxicity and neuroinflammation in Alzheimer’s disease. Front. Neurosci..

[49] Kunkle BW (2019). Genetic meta-analysis of diagnosed Alzheimer’s disease identifies new risk loci and implicates Aβ, tau, immunity and lipid processing. Nat. Genet..

[50] Noori A, Mezlini AM, Hyman BT, Serrano-Pozo A, Das S (2021). Systematic review and meta-analysis of human transcriptomics reveals neuroinflammation, deficient energy metabolism, and proteostasis failure across neurodegeneration. Neurobiol. Dis..

[51] Herdewyn S (2014). Prevention of intestinal obstruction reveals progressive neurodegeneration in mutant TDP-43 (A315T) mice. Mol. Neurodegener..

[52] Congdon EE, Sigurdsson EM (2018). Tau-targeting therapies for Alzheimer disease. Nat. Rev. Neurol..

[53] Shineman DW (2011). Accelerating drug discovery for Alzheimer’s disease: best practices for preclinical animal studies. Alzheimer’s Res. Ther..

[54] Santacruz K (2005). Tau suppression in a neurodegenerative mouse model improves memory function. Science.

[55] Ramsden M (2005). Age-dependent neurofibrillary tangle formation, neuron loss, and memory impairment in a mouse model of human tauopathy (P301L). J. Neurosci..

[56] Yoshiyama Y (2007). Synapse loss and microglial activation precede tangles in a P301S tauopathy mouse model. Neuron.

[57] Goodwin LO (2019). Large-scale discovery of mouse transgenic integration sites reveals frequent structural variation and insertional mutagenesis. Genome Res..

[58] Gamache J (2019). Factors other than hTau overexpression that contribute to tauopathy-like phenotype in rTg4510 mice. Nat. Commun..

[59] Iba M (2013). Synthetic tau fibrils mediate transmission of neurofibrillary tangles in a transgenic mouse model of Alzheimer’s-like tauopathy. J. Neurosci..

[60] Boluda S (2015). Differential induction and spread of tau pathology in young PS19 tau transgenic mice following intracerebral injections of pathological tau from Alzheimer’s disease or corticobasal degeneration brains. Acta Neuropathol..

[61] Woerman AL (2017). Kinetics of human mutant tau prion formation in the brains of 2 transgenic mouse lines. JAMA Neurol..

[62] Zhang B (2018). A brain-penetrant triazolopyrimidine enhances microtubule-stability, reduces axonal dysfunction and decreases tau pathology in a mouse tauopathy model. Mol. Neurodegener..

[63] Goedert M, Spillantini MG, Cairns NJ, Crowther RA (1992). Tau proteins of alzheimer paired helical filaments: abnormal phosphorylation of all six brain isoforms. Neuron.

[64] Taniguchi-Watanabe S (2016). Biochemical classification of tauopathies by immunoblot, protein sequence and mass spectrometric analyses of sarkosyl-insoluble and trypsin-resistant tau. Acta Neuropathol..

[65] Ising C (2019). NLRP3 inflammasome activation drives tau pathology. Nature.

[66] Stancu I-C (2019). Aggregated Tau activates NLRP3–ASC inflammasome exacerbating exogenously seeded and non-exogenously seeded Tau pathology in vivo. Acta Neuropathol..

[67] Panda C (2021). Aggregated tau-PHF6 (VQIVYK) potentiates NLRP3 inflammasome expression and autophagy in human microglial cells. Cells.

[68] Croft CL (2021). Photodynamic studies reveal rapid formation and appreciable turnover of tau inclusions. Acta Neuropathol..

[69] Rocher AB (2010). Structural and functional changes in tau mutant mice neurons are not linked to the presence of NFTs. Exp. Neurol..

[70] Kuchibhotla KV (2014). Neurofibrillary tangle-bearing neurons are functionally integrated in cortical circuits in vivo. Proc. Natl Acad. Sci. USA.

[71] Buée L (2010). From tau phosphorylation to tau aggregation: what about neuronal death?. Biochemical. Soc. Trans..

[72] Andorfer C (2003). Hyperphosphorylation and aggregation of tau in mice expressing normal human tau isoforms. J. Neurochem..

[73] Alonso AdC, Zaidi T, Novak M, Grundke-Iqbal I, Iqbal K (2001). Hyperphosphorylation induces self-assembly of into tangles of paired helical filaments/straight filaments. Proc. Natl Acad. Sci. USA.

[74] Del C. Alonso A, Grundke-Iqbal I, Iqbal K (1996). Alzheimer’s disease hyperphosphorylated tau sequesters normal tau into tangles of filaments and disassembles microtubules. Nat. Med..

[75] Narasimhan S (2017). Pathological tau strains from human brains recapitulate the diversity of tauopathies in nontransgenic mouse brain. J. Neurosci..

[76] Guo JL (2016). Unique pathological tau conformers from Alzheimer’s brains transmit tau pathology in nontransgenic mice. J. Exp. Med..

[77] Strang KH, Golde TE, Giasson BI (2019). MAPT mutations, tauopathy, and mechanisms of neurodegeneration. Lab. Invest..

[78] Aoyagi H, Hasegawa M, Tamaoka A (2007). Fibrillogenic nuclei composed of P301L mutant tau induce elongation of P301L tau but not wild-type tau. J. Biol. Chem..

[79] Miyasaka T (2001). Selective deposition of mutant tau in the FTDP-17 brain affected by the P301L mutation. J. Neuropathol. Exp. Neurol..

[80] Spillantini MG, Crowther RA, Kamphorst W, Heutink P, Van Swieten JC (1998). Tau pathology in two Dutch families with mutations in the microtubule- binding region of tau. Am. J. Pathol..

[81] Bi F (2013). Reactive astrocytes secrete lcn2 to promote neuron death. Proc. Natl Acad. Sci. USA.

[82] Staurenghi, E. et al. Oxysterols present in Alzheimer’s disease brain induce synaptotoxicity by activating astrocytes: a major role for lipocalin-2. *Redox Biol*. **39**, 101837 (2021). 10.1016/j.redox.2020.101837 PMC7772793 33360775

[83] Choi J, Lee H-W, Suk K (2011). Increased plasma levels of lipocalin 2 in mild cognitive impairment. J. Neurol. Sci..

[84] Naudé PJW (2012). Lipocalin 2: novel component of proinflammatory signaling in Alzheimer’s disease. FASEB J..

[85] Papassotiropoulos A (2006). Cholesterol 25-hydroxylase on chromosome 10q is a susceptibility gene for sporadic alzheimer’s disease. Neurodegener. Dis..

[86] Bhatia S, Kim WS, Shepherd CE, Halliday GM (2019). Apolipoprotein D upregulation in Alzheimer’s disease but not frontotemporal dementia. J. Mol. Neurosci..

[87] Terrisse L (2002). Increased levels of apolipoprotein D in cerebrospinal fluid and hippocampus of Alzheimer’s patients. J. Neurochem..

[88] Miyashita, A. et al. Genes associated with the progression of neurofibrillary tangles in Alzheimer’s disease. *Transl. Psychiatry* **4**, e396 (2014). 10.1038/tp.2014.35 PMC4080317 26126179

[89] Clarke CM (2007). Visceral neuropathy and intestinal pseudo-obstruction in a murine model of a nuclear inclusion disease. Gastroenterology.

[90] Esmaeili MA, Panahi M, Yadav S, Hennings L, Kiaei M (2013). Premature death of TDP-43 (A315T) transgenic mice due to gastrointestinal complications prior to development of full neurological symptoms of amyotrophic lateral sclerosis. Int. J. Exp. Pathol..

[91] Zhang, T. et al. Comparative epidemiological investigation of Alzheimer’s disease and colorectal cancer: the possible role of gastrointestinal conditions in the pathogenesis of AD. *Front. Aging Neurosci*. **10**, 176 (2018). 10.3389/fnagi.2018.00176 PMC6172982 30323761

[92] Fu P, Gao M, Yung KKL (2020). Association of intestinal disorders with Parkinson’s disease and Alzheimer’s disease: a systematic review and meta-analysis. ACS Chem. Neurosci..

[93] Prigent A (2020). Tau accumulates in Crohn’s disease gut. FASEB J..

[94] Dickinson ME (2016). High-throughput discovery of novel developmental phenotypes. Nature.

[95] Petry FR (2014). Specificity of anti-tau antibodies when analyzing mice models of Alzheimer’s disease: problems and solutions. PLoS ONE.

[96] Duff K (2000). Characterization of pathology in transgenic mice over-expressing human genomic and cDNA tau transgenes. Neurobiol. Dis..

[97] Dhillon J-KS (2017). A novel panel of α-synuclein antibodies reveal distinctive staining profiles in synucleinopathies. PLoS ONE.

[98] Vallet PG (1992). A comparative study of histological and immunohistochemical methods for neurofibrillary tangles and senile plaques in Alzheimer’s disease. Acta Neuropathol..

[99] Giasson BI (2002). Neuronal alpha-synucleinopathy with severe movement disorder in mice expressing A53T human alpha-synuclein. Neuron.

[100] Rodríguez EM, Yulis R, Peruzzo B, Alvial G, Andrade R (1984). Standardization of various applications of methacrylate embedding and silver methenamine for light and electron microscopy immunocytochemistry. Histochemistry.

[101] Kuo Y-M (2010). Extensive enteric nervous system abnormalities in mice transgenic for artificial chromosomes containing Parkinson disease-associated α-synuclein gene mutations precede central nervous system changes. Hum. Mol. Genet..

