# Supplementary material for: Pathogenic tau recruits wild-type tau into brain inclusions and induces gut degeneration in transgenic SPAM mice
Source: Commun Biol. 2022 May 12;5:446. doi: 10.1038/s42003-022-03373-1 (PMC9098443; doi:10.1038/s42003-022-03373-1)

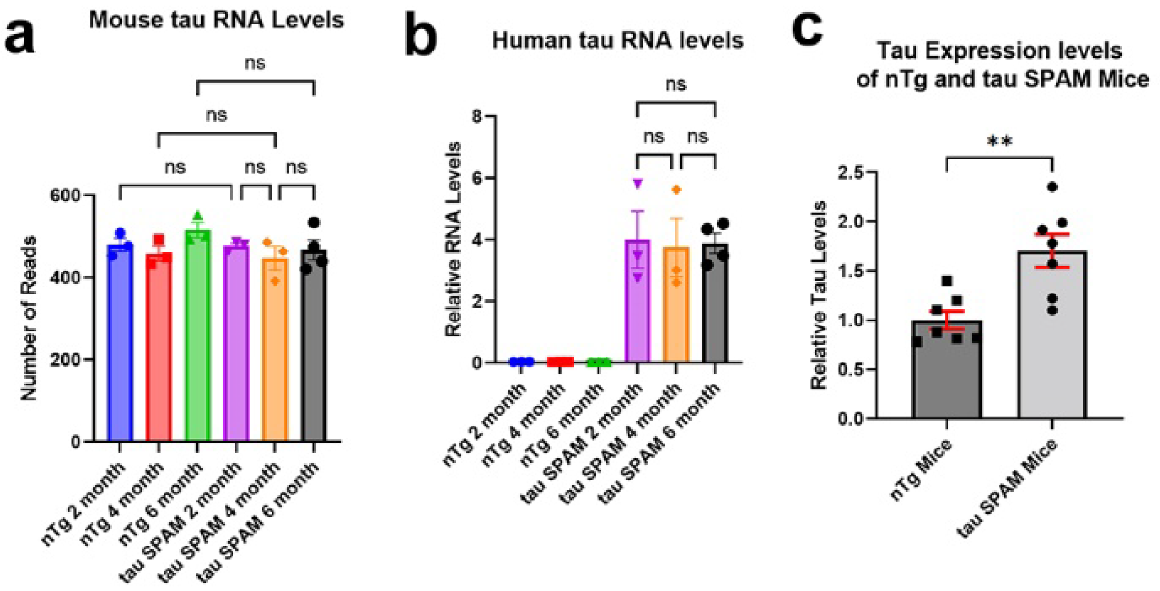


**Supplemental Figure 1**. **Human tau RNA levels are comparable between different ages of tau SPAM mice.** (**a**) Relative tau RNA levels were collected from RNA-sequencing transcriptomic. Mouse tau RNA levels (number of reads) are comparable between nTg and tau SPAM mice at different ages of 2, 4, and 6 months for both sexes. ns = not statistically significant. (**b**). Human tau RNA levels are not significantly different in tau SPAM mice at different ages of 2, 4, and 6 months for both sexes. Levels are relative to the average number of reads for mouse tau. ns = not statistically significant. (C) Total tau expression levels were quantified based on western blots of 2 month old nTg and tau SPAM mice using a total tau antibody (3026) and either actin or GAPDH antibodies as loading controls. Relative tau expression in tau SPAM mice is about 1.7X of nTg mice (N = 7) after normalizing to loading control. Error bars represent standard errors of the mean. ** = $p < 0.01$.

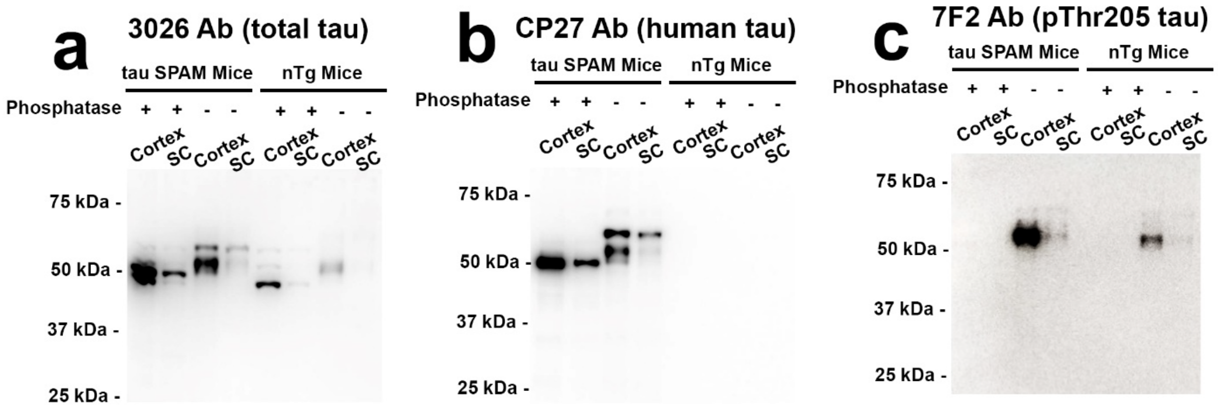


**Supplemental Figure 2**. **In vitro dephosphorylation of tau in lysates**. Brain cortex and spinal cord (SC) lysates from 6-month-old nTg and tau SPAM mice were incubated with (+) or without (-) phosphatase and resolved by SDS-PAGE following by immunoblotting blotting with (**a**) antibody 3026 against total tau showing both mouse and human tau, (**b**) antibody CP27 specific for human tau and (**c**) antibody 7F2 specific for tau pThr205. The loss of 7F2 reactivity confirms that tau was dephosphorylated with phosphatase treatment that results in an increase in the mobility of tau closer to its predicted naïve molecular mass. The relative molecular mass markers are shown on the left.

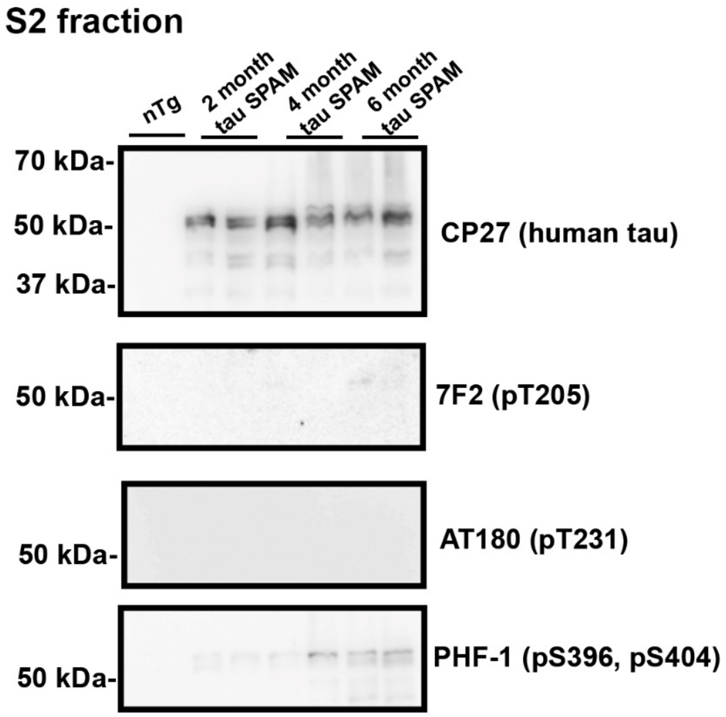


**Supplemental Figure 3**. **Tau in the S2 brain cortical fraction from tau SPAM mice at 2, 4, and 6 months of age.** The S2 fractions represent soluble fractions after extraction with Triton-X-100 detergent. The fractions were resolved by SDS-PAGE and assessed by immunoblotting with CP27 antibody specific for human tau and tau phospho-specific antibody 7F2, (pThr205), AT180 (pThr231), and PHF-1 (pSer396/pSer404). The relative molecular mass markers are shown on the left.

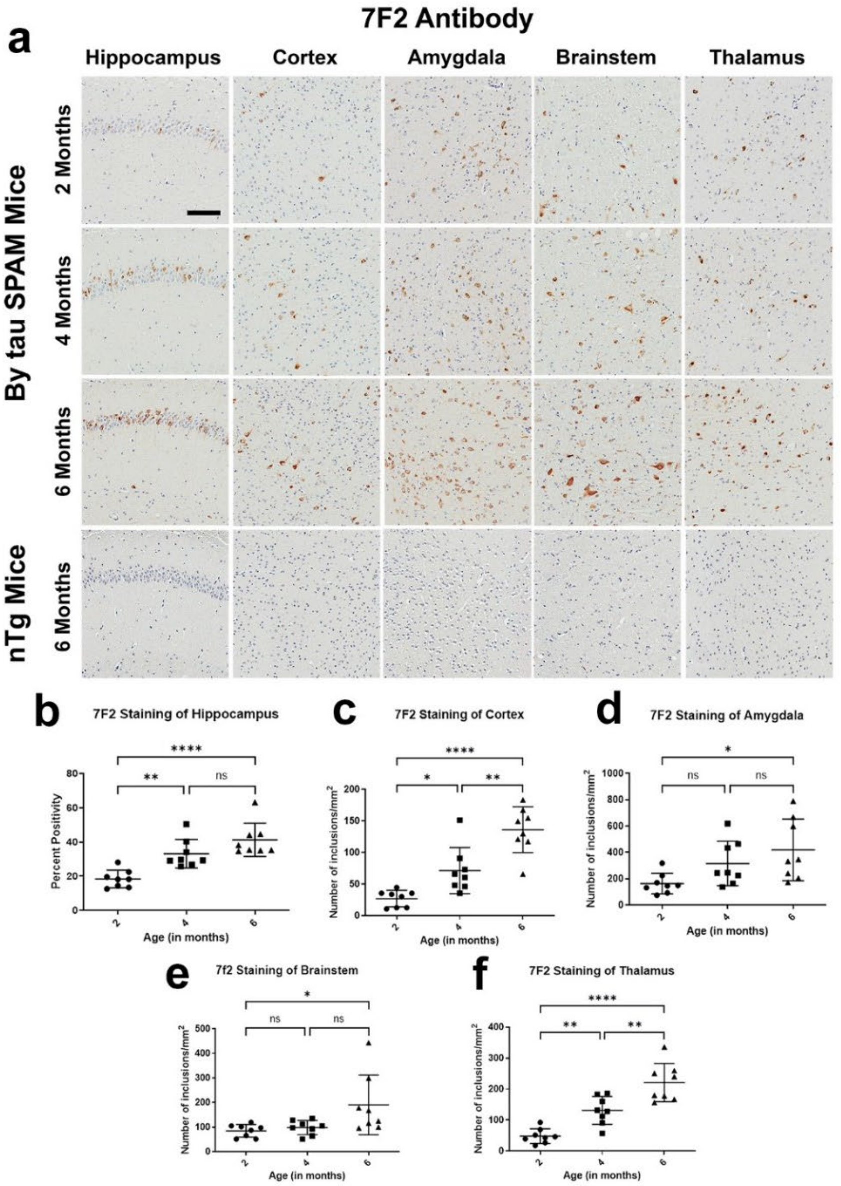


**Supplemental Figure 4**. **7F2-positive phosphorylated tau inclusions progressively increase with age in tau SPAM mice.** (**a**) Brain samples from tau SPAM mice at ages 2, 4, and 6 months were stained by IHC with 7F2 antibody specific for tau phosphorylated at T205. Tau inclusions were abundantly found in multiple brain areas as early as 2 months but further increase with age. Scale bar = 100 μm. Tau inclusions progressively increased in the (**b**) hippocampus, (**c**) cortex, (**d**) amygdala, (**e**) brainstem and (**f**) thalamus of 2, 4, and, 6 month old mice (N = 8, 4M, 4F for each time point). Pathology was quantified with Aperio algorithms and compared using one-way ANOVA with Dunnett's test. v **** = $p < 0.0001$, ** = $p < 0.01$, * = $p < 0.05$, ns = not statistically significant.

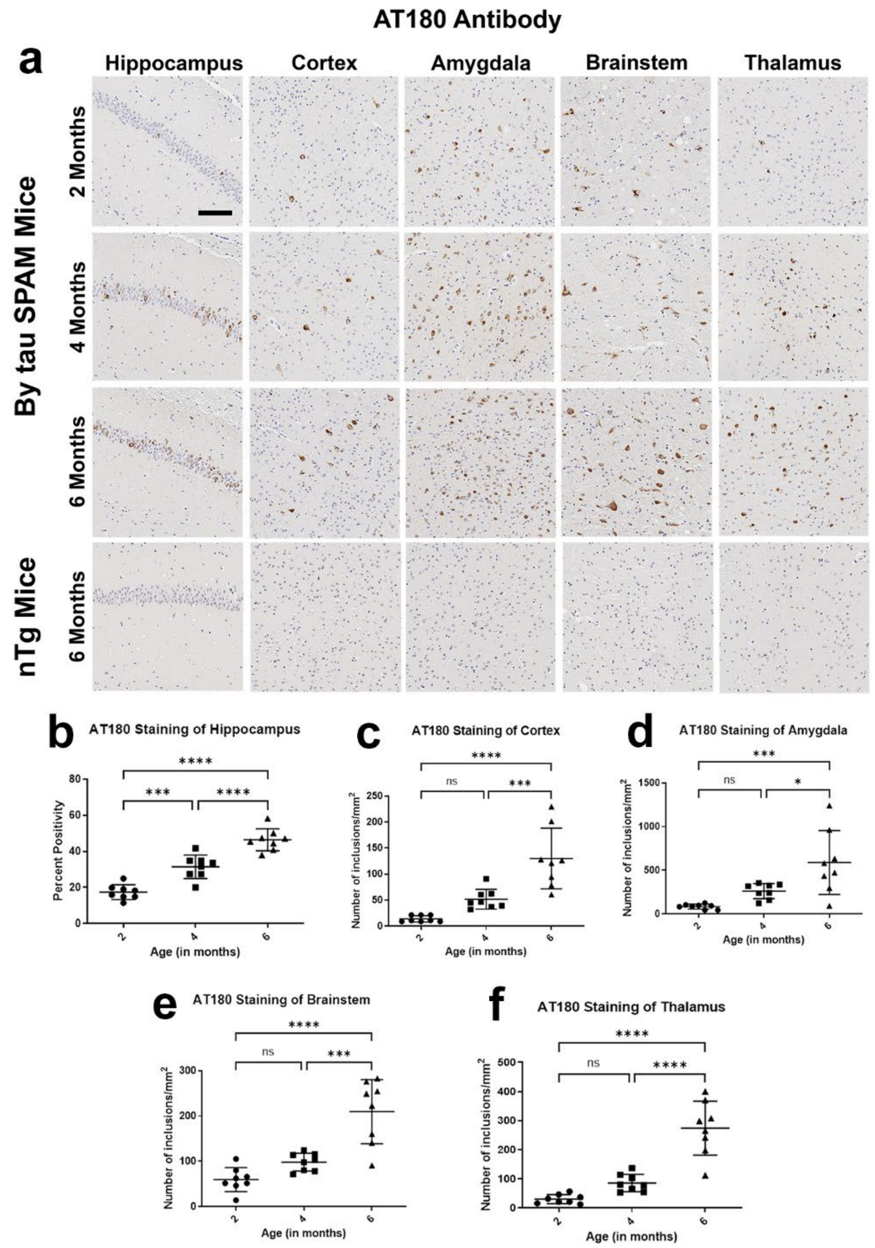


**Supplemental Figure 5. AT180-positive phosphorylated tau inclusions progressively increase with age in tau SPAM mice.** (**a**) Brain samples from tau SPAM mice at ages 2, 4, and 6 months were stained with by IHC with AT180 antibody specific for tau phosphorylated at T231. Tau inclusions were abundantly found in multiple brain areas as early as 2 months but further increase with age. Scale bar = 100 μm. Tau inclusions progressively increased in the (**b**) hippocampus, (**c**) cortex, (**d**) amygdala, (**e**) brainstem and (**f**) thalamus of 2, 4, and 6-month-old mice (N = 8, 4M, 4F for each time point). Pathology was quantified with Aperio algorithms and compared using one-way ANOVA with Dunnett's test. Error bars represent standard errors of the mean. **** = $p < 0.0001$, ** = $p < 0.01$, * = $p < 0.05$, ns = not statistically significant.

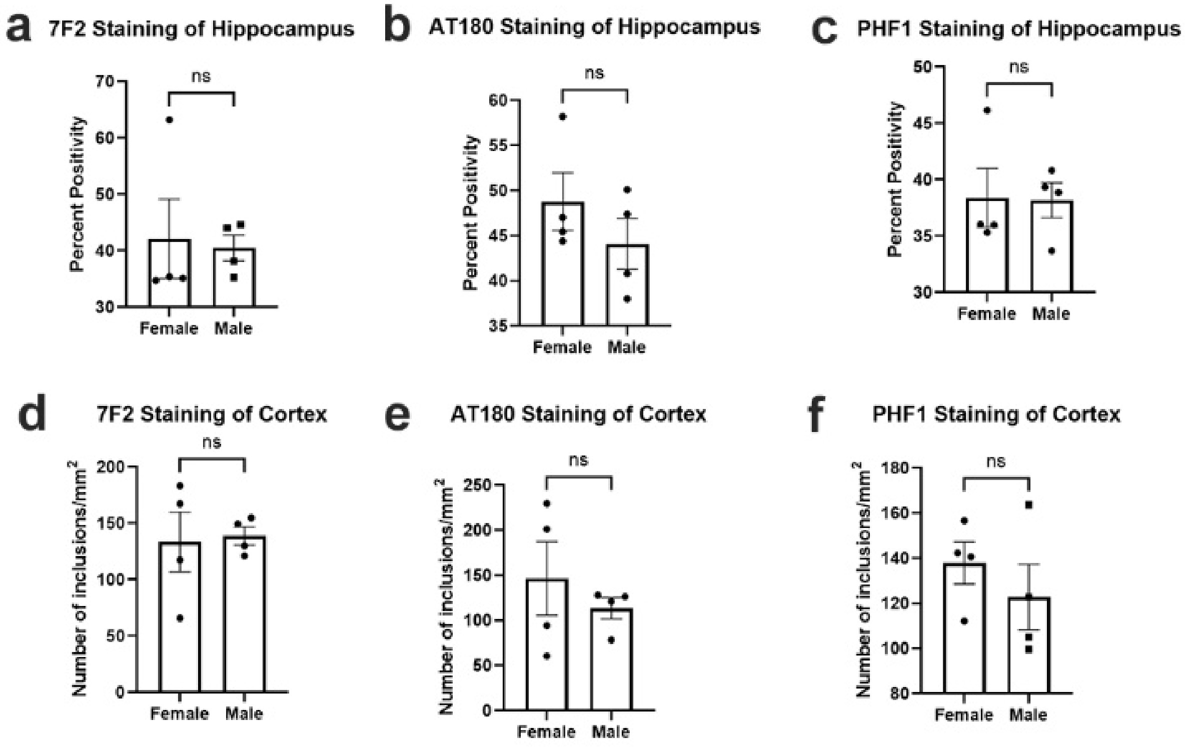


**Supplemental Figure 6. Tau phosphorylation and inclusion burden reach a similar level in both males and females**. Tau phosphorylation-specific antibodies (7F2, AT180 and PHF1) were used to stain cortex and hippocampus of tau SPAM mice at 6 months. There were no significant differences in tau inclusion burden stained with antibodies s7F2 (**a, d**), AT180 (**b, e**), and PHF1 (**c, f**) in hippocampus and cortex. Error bars represent standard errors of the mean. ns = not statistically significant.

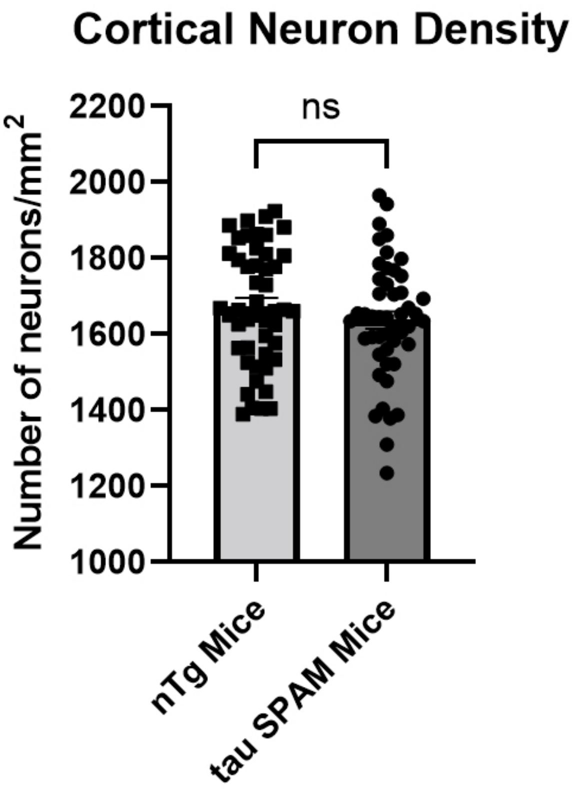


**Supplemental Figure 7**. **Neuronal loss is not observed in tau SPAM mice at 6 months in the cortex.** Random sections were stained with NeuN antibody to detect neuronal nuclei and quantified with Aperio algorithms. Cortical neuron density was not statistically different between tau SPAM mice and nTg mice at 6 months (two-tailed t test, t = 1.118, df = 94, p = 0.9654) for N = 48 different sections from N = 8 mice in each group. Error bars represent standard errors of the mean. ns = not statistically significant.

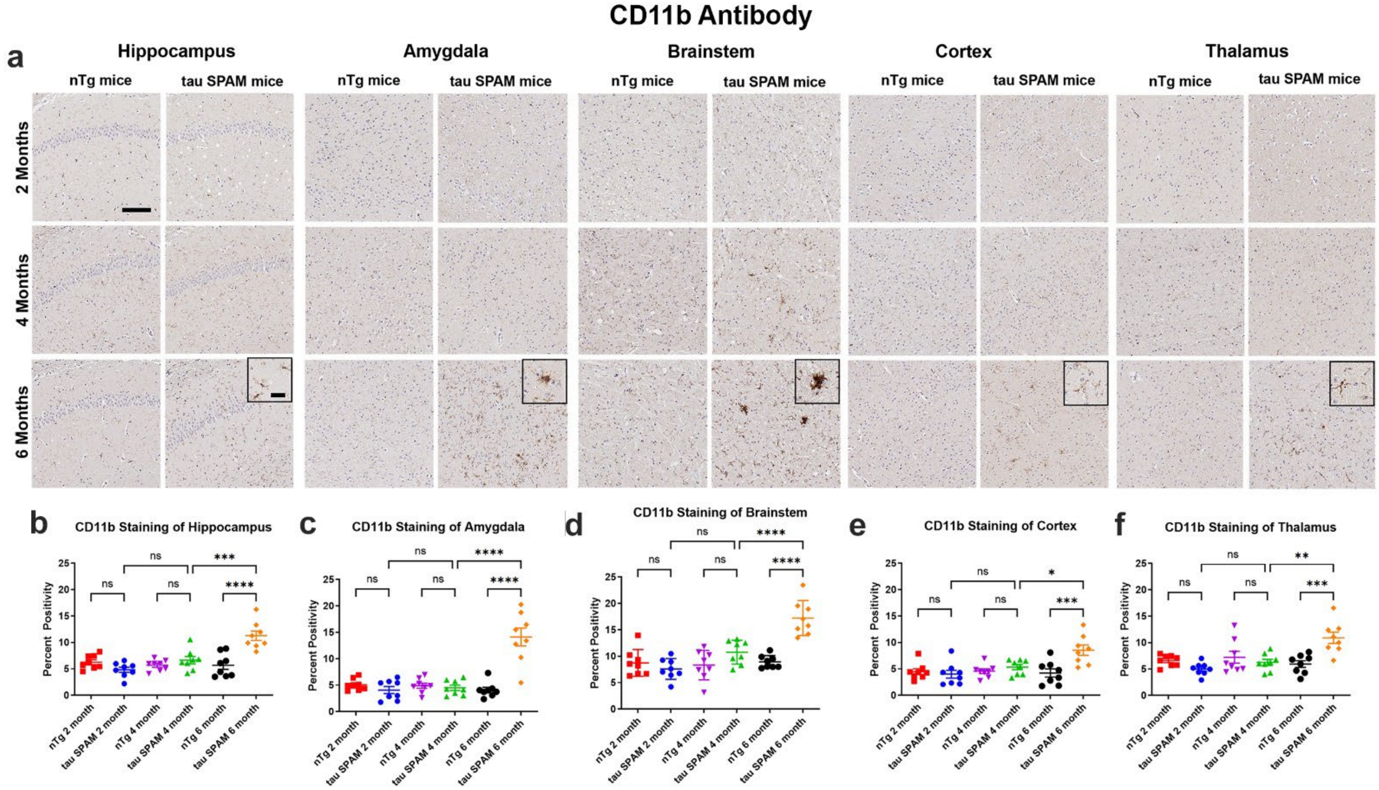


**Supplemental Figure 8. Microgliosis begins at 6 months in nearly all brain areas with tau pathology in tau SPAM mice.** (**a**) Anti-CD11b IHC was used to stain brain samples from tau SPAM mice and nTg mice at ages 2, 4, and 6 months. CD11b immunoreactivity was significantly increased in the (**b**) hippocampus, (**c**) amygdala, (**d**) brainstem, (**e**) cortex and (**f**) thalamus at 6 months (N = 8, 4M, 4F for each time point). Scale bar = 100 μm for main figure and 50 μm for insert. Staining was quantified with Aperio algorithms and compared using one-way ANOVA with Dunnett's test. Error bars represent standard errors of the mean. **** = $p < 0.0001$, *** = $p < 0.001$, ** = $p < 0.01$, * = $p < 0.05$, ns = not statistically significant.

# Figure 1B and 1C

3026

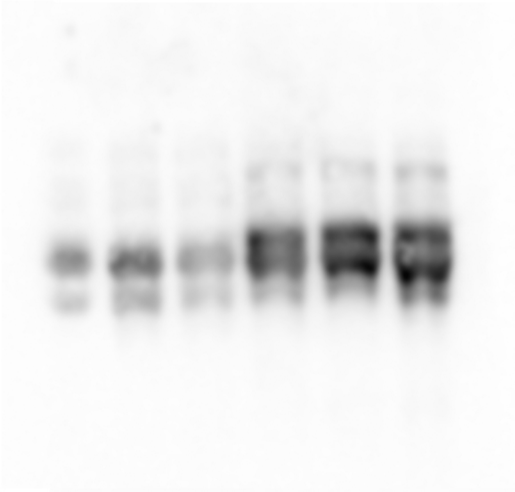

actin

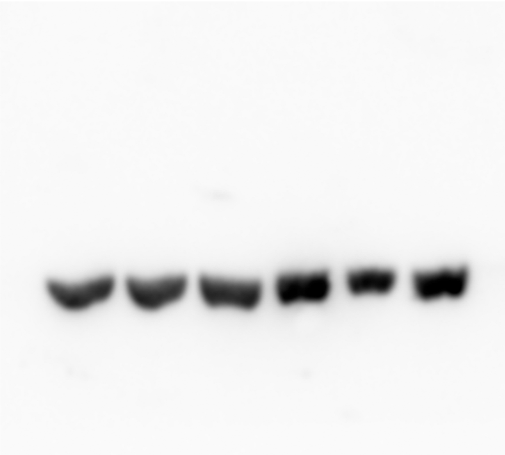

CP27

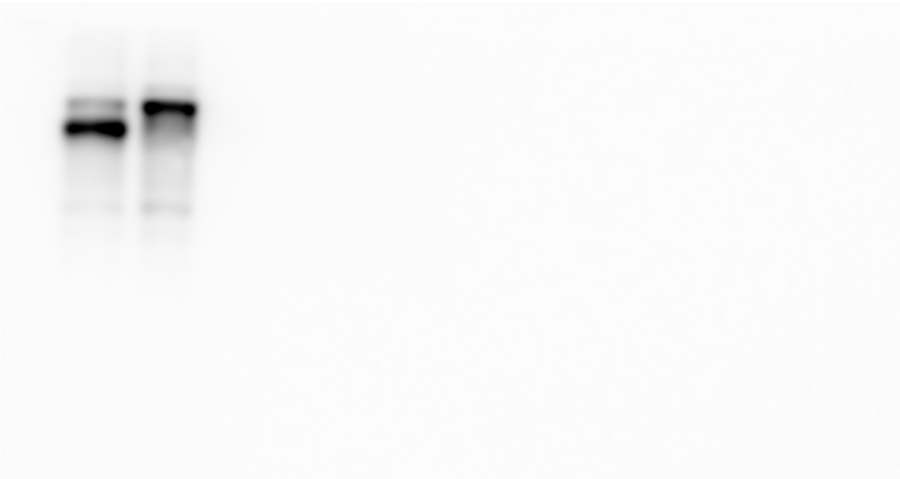

# Figure 3B

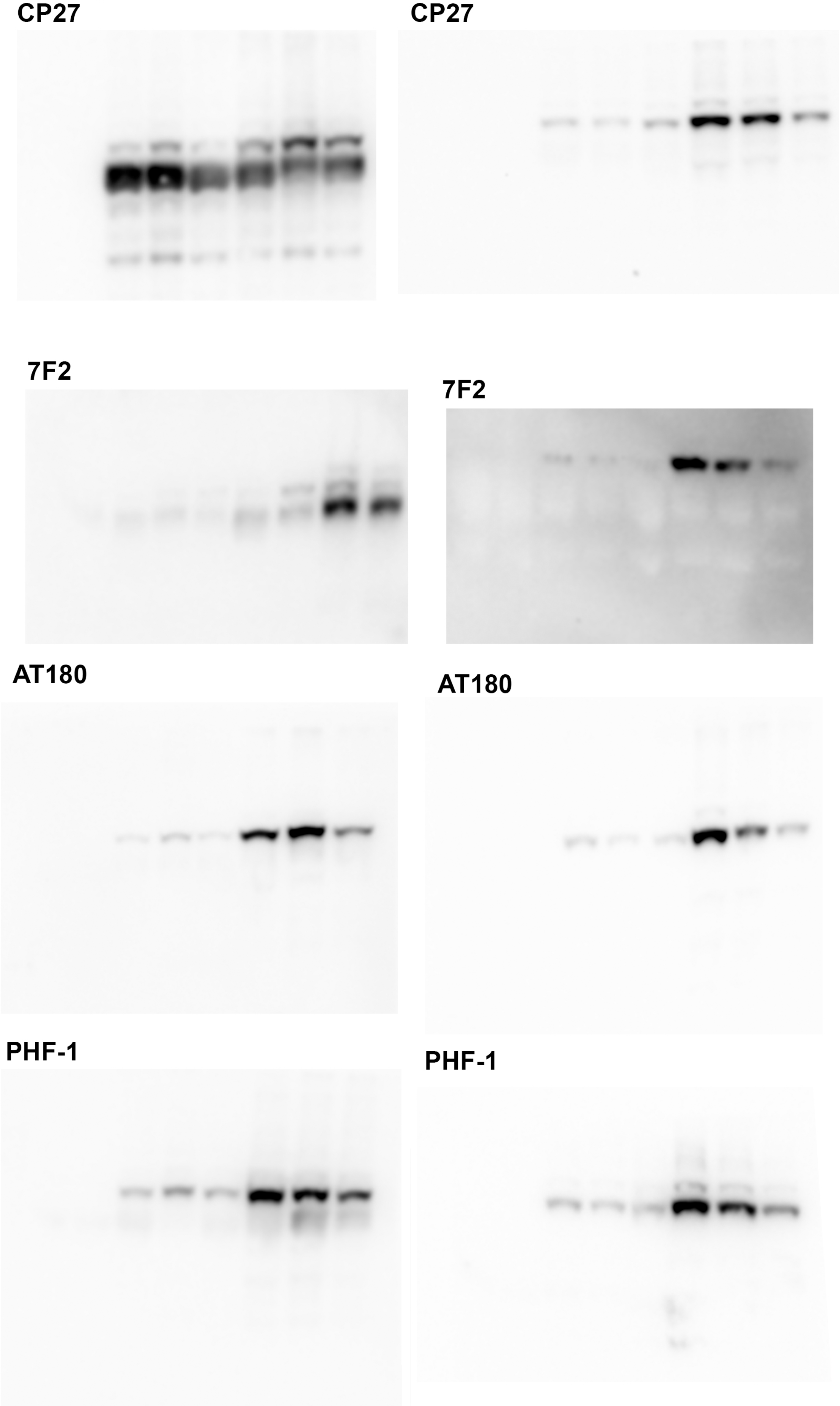

# Figure 3C

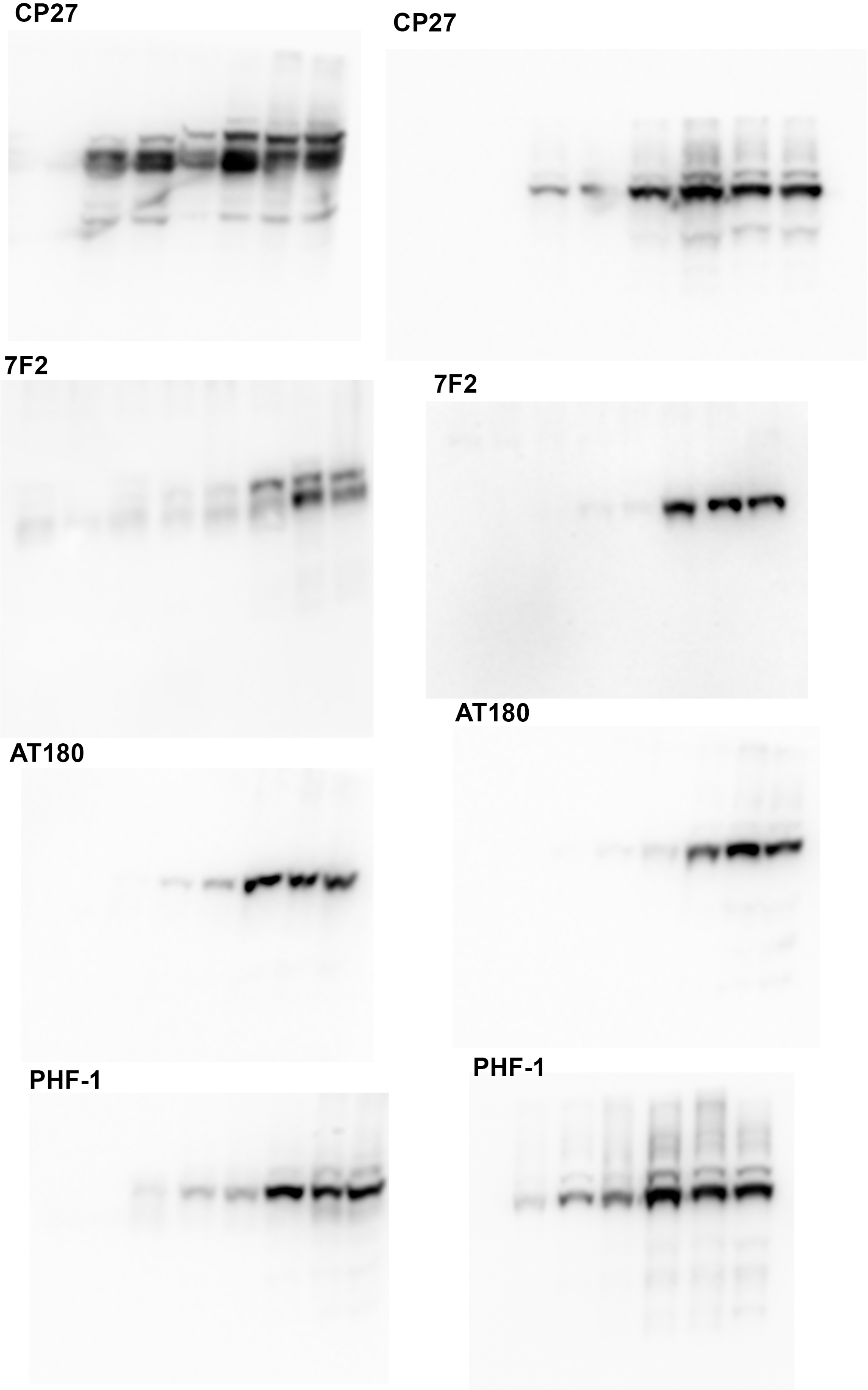

# Figure 6G

T49

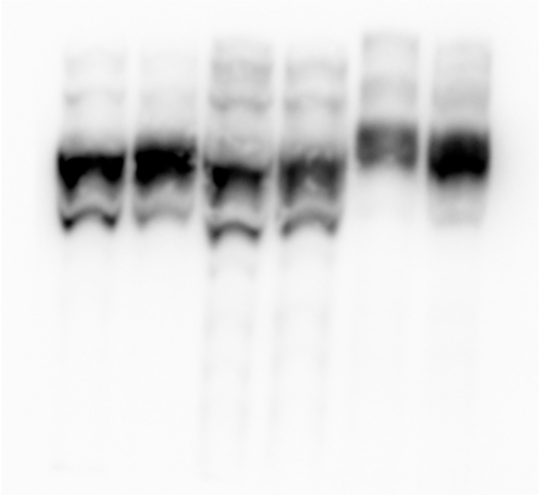

T49

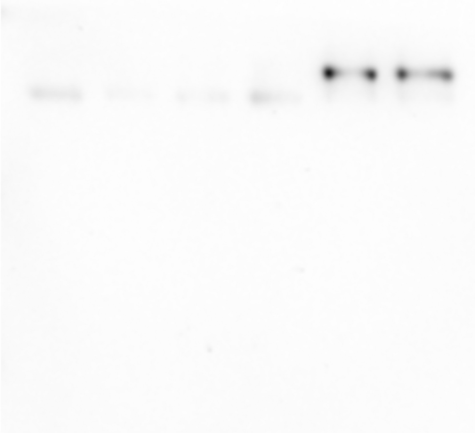

# Figure 10A

CP27

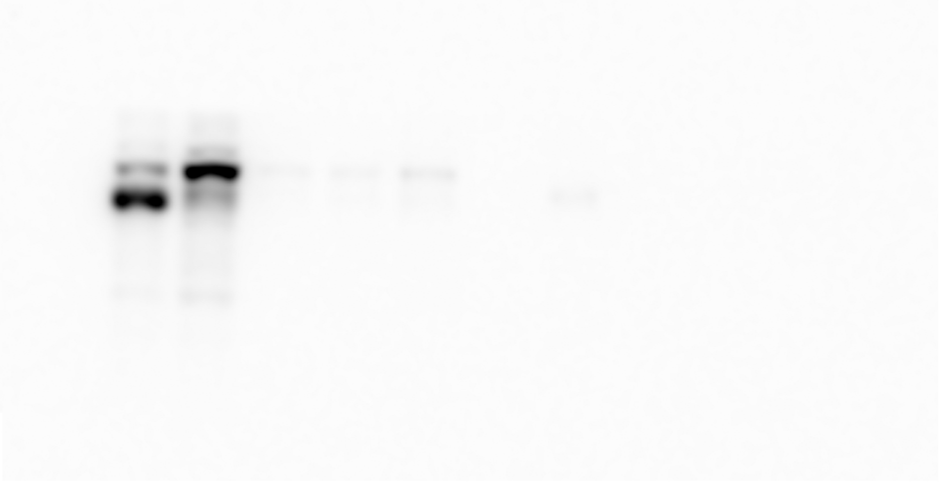

CP27

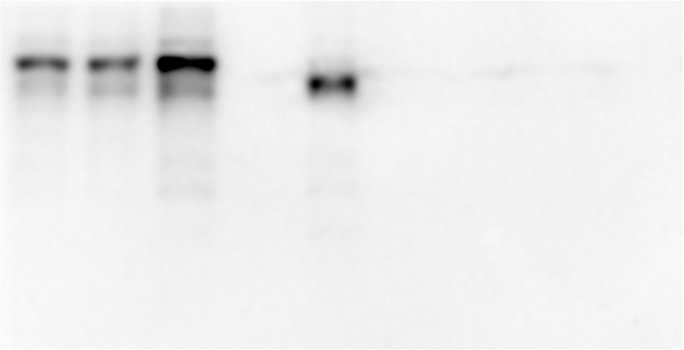

Supplement: Supplementary file 1 — Supplemental Information [file 42003_2022_3373_MOESM1_ESM.pdf]
