# Supplementary material for: Pathogenic tau recruits wild-type tau into brain inclusions and induces gut degeneration in transgenic SPAM mice
Source: Commun Biol. 2022 May 12;5:446. doi: 10.1038/s42003-022-03373-1 (PMC9098443; doi:10.1038/s42003-022-03373-1)
Supplement: Supplementary file 2 — Description of Additional Supplementary Files [file 42003_2022_3373_MOESM2_ESM.pdf]

Description of Additional Supplementary Files

File name: Supplementary Data 1
Description: Source data underlying Figures 4, 7, 8, 9 and 10.
