# Supplementary material for: Pathogenic tau recruits wild-type tau into brain inclusions and induces gut degeneration in transgenic SPAM mice
Source: Commun Biol. 2022 May 12;5:446. doi: 10.1038/s42003-022-03373-1 (PMC9098443; doi:10.1038/s42003-022-03373-1)
Supplement: Supplementary file 4 — Reporting Summary [file 42003_2022_3373_MOESM4_ESM.pdf]

nature portfolio

Corresponding author(s): COMMSBIO-21-3406B

Last updated by author(s): 2022-4-1

Reporting Summary

Nature Portfolio wishes to improve the reproducibility of the work that we publish. This form provides structure for consistency and transparency in reporting. For further information on Nature Portfolio policies, see our Editorial Policies and the Editorial Policy Checklist.

Statistics

For all statistical analyses, confirm that the following items are present in the figure legend, table legend, main text, or Methods section.

n/a	Confirmed	
☐	☒	The exact sample size (n) for each experimental group/condition, given as a discrete number and unit of measurement
☐	☒	A statement on whether measurements were taken from distinct samples or whether the same sample was measured repeatedly
☐	☒	The statistical test(s) used AND whether they are one- or two-sided<br>*Only common tests should be described solely by name; describe more complex techniques in the Methods section.*
☐	☒	A description of all covariates tested
☒	☐	A description of any assumptions or corrections, such as tests of normality and adjustment for multiple comparisons
☐	☒	A full description of the statistical parameters including central tendency (e.g. means) or other basic estimates (e.g. regression coefficient) AND variation (e.g. standard deviation) or associated estimates of uncertainty (e.g. confidence intervals)
☒	☐	For null hypothesis testing, the test statistic (e.g. F, t, r) with confidence intervals, effect sizes, degrees of freedom and P value noted<br>*Give P values as exact values whenever suitable.*
☒	☐	For Bayesian analysis, information on the choice of priors and Markov chain Monte Carlo settings
☒	☐	For hierarchical and complex designs, identification of the appropriate level for tests and full reporting of outcomes
☒	☐	Estimates of effect sizes (e.g. Cohen's d, Pearson's r), indicating how they were calculated

Our web collection on statistics for biologists contains articles on many of the points above.

Software and code

Policy information about availability of computer code

Data collection	N/A
Data analysis	Software used include Partek Flow, Graphpad Prism Version 9.0.2

For manuscripts utilizing custom algorithms or software that are central to the research but not yet described in published literature, software must be made available to editors and reviewers. We strongly encourage code deposition in a community repository (e.g. GitHub). See the Nature Portfolio guidelines for submitting code & software for further information.

Data

Policy information about availability of data

All manuscripts must include a data availability statement. This statement should provide the following information, where applicable:

- Accession codes, unique identifiers, or web links for publicly available datasets
- A description of any restrictions on data availability
- For clinical datasets or third party data, please ensure that the statement adheres to our policy

The data that support the findings of this study are available as supplemental files or from the corresponding author upon reasonable request.

March 2021

Field-specific reporting

Please select the one below that is the best fit for your research. If you are not sure, read the appropriate sections before making your selection.

☒ Life sciences ☐ Behavioural & social sciences ☐ Ecological, evolutionary & environmental sciences

For a reference copy of the document with all sections, see nature.com/documents/nr-reporting-summary-flat.pdf

Life sciences study design

All studies must disclose on these points even when the disclosure is negative.

Sample size	Since we characterized a new mouse model, it wasn't possible to make a definitive power analysis. However, we included sufficient n numbers for each experiment based on previous data on tau transgenic mice.
Data exclusions	For the data set on enteric neuron counts, one data point was excluded based on outlier test described in Materials and Methods.
Replication	All results were replicated.
Randomization	For enteric neuron counts, all mice were randomized with a new number identified.
Blinding	For enteric neuron counts, all three raters were blinded to the genotype.

Reporting for specific materials, systems and methods

We require information from authors about some types of materials, experimental systems and methods used in many studies. Here, indicate whether each material, system or method listed is relevant to your study. If you are not sure if a list item applies to your research, read the appropriate section before selecting a response.

Materials & experimental systems

n/a	Involved in the study	
☐	☒	Antibodies
☒	☐	Eukaryotic cell lines
☒	☐	Palaeontology and archaeology
☐	☒	Animals and other organisms
☒	☐	Human research participants
☒	☐	Clinical data
☒	☐	Dual use research of concern

Methods

n/a	Involved in the study	
☒	☐	ChIP-seq
☒	☐	Flow cytometry
☒	☐	MRI-based neuroimaging

Antibodies

Antibodies used	Antibodies have been listed in Materials and Methods. Further details can be provided if requested.
Validation	Antibodies were validated by manufacturer (e.g. AT180 antibody from Thermo Fisher) or by previous publications from our lab (e.g. 7F2 antibody).

Animals and other organisms

Policy information about studies involving animals; ARRIVE guidelines recommended for reporting animal research

Laboratory animals	New tau SPAM mice are lab mice (Mus Musculus) with a C3H/BL6 background and expresses pathogenic tau with P301S and S320F mutations (SPAM). This includes both sexes and aged to 6 months. Litter mates were nontransgenic mice (Mus Musculus) with the same C3H/BL6 background.
Wild animals	N/A
Field-collected samples	N/A
Ethics oversight	IACUC at University of Florida

Note that full information on the approval of the study protocol must also be provided in the manuscript.
